# Homeolog loss and expression changes in natural populations of the recently and repeatedly formed allotetraploid *Tragopogon mirus *(Asteraceae)

**DOI:** 10.1186/1471-2164-11-97

**Published:** 2010-02-08

**Authors:** Jin Koh, Pamela S Soltis, Douglas E Soltis

**Affiliations:** 1 Department of Biology, University of Florida, Gainesville, Florida, 32611 USA; 2 Florida Museum of Natural History, University of Florida, Gainesville, Florida, 32611 USA

## Abstract

**Background:**

Although polyploidy has long been recognized as a major force in the evolution of plants, most of what we know about the genetic consequences of polyploidy comes from the study of crops and model systems. Furthermore, although many polyploid species have formed repeatedly, patterns of genome evolution and gene expression are largely unknown for natural polyploid populations of independent origin. We therefore examined patterns of loss and expression in duplicate gene pairs (homeologs) in multiple individuals from seven natural populations of independent origin of *Tragopogon mirus *(Asteraceae), an allopolyploid that formed repeatedly within the last 80 years from the diploids *T. dubius *and *T. porrifolius*.

**Results:**

Using cDNA-AFLPs, we found differential band patterns that could be attributable to gene silencing, novel expression, and/or maternal/paternal effects between *T. mirus *and its diploid parents. Subsequent cleaved amplified polymorphic sequence (CAPS) analyses of genomic DNA and cDNA revealed that 20 of the 30 genes identified through cDNA-AFLP analysis showed additivity, whereas nine of the 30 exhibited the loss of one parental homeolog in at least one individual. Homeolog loss (versus loss of a restriction site) was confirmed via sequencing. The remaining gene (*ADENINE-DNA GLYCOSYLASE*) showed ambiguous patterns in *T. mirus *because of polymorphism in the diploid parent *T. dubius*. Most (63.6%) of the homeolog loss events were of the *T. dubius *parental copy. Two genes, *NUCLEAR RIBOSOMAL DNA *and *GLYCERALDEHYDE-3-PHOSPHATE DEHYDROGENASE*, showed differential expression of the parental homeologs, with the *T. dubius *copy silenced in some individuals of *T. mirus*.

**Conclusions:**

Genomic and cDNA CAPS analyses indicated that plants representing multiple populations of this young natural allopolyploid have experienced frequent and preferential elimination of homeologous loci. Comparable analyses of synthetic F_1 _hybrids showed only additivity. These results suggest that loss of homeologs and changes in gene expression are not the immediate result of hybridization, but are processes that occur following polyploidization, occurring during the early (<40) generations of the young polyploid. Both *T. mirus *and a second recently formed allopolyploid, *T. miscellus*, exhibit more homeolog losses than gene silencing events. Furthermore, both allotetraploids undergo biased loss of homeologs contributed by their shared diploid parent, *T. dubius*. Further studies are required to assess whether the results for the 30 genes so far examined are representative of the entire genome.

## Background

Polyploidy is a particularly important evolutionary mechanism in flowering plants [1-4]. During the past 70 years, many plant biologists have estimated the frequency of polyploidy in the angiosperms using analysis of base chromosome numbers [5-8], as well as measurements of stomatal size in fossil and extant taxa [9]. Based on these approaches, researchers estimated that from 40% to 70% of angiosperms have experienced polyploidy in their evolutionary history [5-7,9]. Recent genomic studies indicate, however, that polyploidy is even more prevalent in angiosperm lineages than previously suspected. Sequencing of the entire nuclear genome of *Arabidopsis thaliana *indicated two or three rounds of genome-wide duplication [10-17]. Complete genome sequences also indicate multiple ancient polyploidy events in *Populus trichocarpa *and *Vitis vinifera *[18-20]. Genomic data (including analyses of ESTs) indicate ancient polyploidy for other angiosperms [21], including the basal angiosperm *Nuphar advena*, the magnoliids *Persea americana*, *Liriodendron tulipifera*, and *Saruma henryi*, the basal monocot *Acorus americanus*, and the basal eudicot *Eschscholzia californica *[22]. It now appears that all angiosperms may have undergone at least one round of genome duplication (reviewed in [23,24]).

Several outcomes for duplicated genes are possible at the genomic and transcriptional levels. First, both members of a duplicate gene pair may retain their original function. Second, one copy of a duplicate gene pair may retain the original function, but the other copy may become lost or silenced [3,13,14,23-26]. Third, duplicate genes may partition the original gene function (subfunctionalization), with one copy active, for example, in one tissue and the other copy active in another tissue [25,27-31]. Fourth, one copy may retain the original function, while the other develops a new function (neofunctionalization) [32-38].

Recent studies have revealed varied consequences of genome evolution and gene expression following polyploidy in diverse angiosperms, including *Arabidopsis *[39-44] and crops such as cotton [31,45,46], wheat [1,47-50], and *Brassica *[51-55]. Several investigations have shown that following polyploidy, rapid genomic rearrangement [48,51,56], gene loss [1,49,53], or gene silencing via DNA methylation [39,41,43,44,49,53] may occur. However, few analyses have explored the genetic and genomic consequences of allopolyploidy in natural systems. Six natural allopolyploids are known to have formed within the past 150 years, thus affording the opportunity to examine the nearly immediate consequences of polyploidization in nature: *Cardamine schulzii *[57], *Senecio cambrensis *[58-63], *Senecio eboracensis *[60], *Spartina anglica *[64-68], and *Tragopogon mirus *and *T. miscellus *[26,51,69-72]. Several studies of these recently formed allopolyploids show evidence of either genomic or expression-level changes, relative to their diploid parents. For example, Salmon et al. [65] showed that methylation patterns differ between the hexaploid parents (*Spartina maritima *and *S. alterniflora*), the independently formed hybrids (*Spartina × townsendii *and *S. × neyrautii*), and the allopolyploid *S. anglica *(formed from *Spartina × townsendii*). In *Senecio*, hybridization of diploid *S. squalidus *with tetraploid *S. vulgaris *forms a sterile triploid, *S*. × *baxteri*, and subsequent genome duplication produced the allohexaploid *S. cambrensis*. Through microarray analysis of floral gene expression patterns in synthetic *S. cambrensis *lines, Hegarty et al. [62,73] observed that the synthetic hybrid *S. × baxteri *showed immediate transcriptional changes compared to the parental expression patterns, and that this "transcriptional shock" was "subsequently calmed" in allohexaploid *S. cambrensis*, suggesting that hybridization and polyploidization have distinct effects on large-scale gene expression in this system.

One of the best systems for the study of naturally occurring polyploids is provided by the genus *Tragopogon *(Asteraceae). *Tragopogon *comprises ca. 100 to 150 species distributed throughout Europe, temperate Asia, and North Africa [74-76]. Three diploid species (*T. dubius*, *T. porrifolius*, and *T. pratensis*) were introduced from Europe into the Palouse region of eastern Washington and adjacent Idaho, USA, in the early 1900s [69,70]. The introduction of these three diploid species brought them into close contact, and as a result, two allotetraploid species (*T. mirus *and *T. miscellus*) formed [69]. First collected in 1949 [69], these recently formed polyploids are less than 80 years old. Morphological, cytological, flavonoid, isozymic, and DNA evidence confirmed the ancestries of these two allotetraploids [77-83]. Multiple lines of evidence suggest that *T. miscellus *has formed recurrently, possibly as many as 21 times, including reciprocal formation, and *T. mirus *has formed repeatedly perhaps 13 times (but not reciprocally) [70,84,85]. Therefore, *T. mirus *and *T. miscellus *afford unique opportunities for the investigation of recent and recurrent polyploid evolution. In fact, nearly every population of these species may have formed independently (V. Symonds et al., unpublished data).

Tate et al. [26,86] and Buggs et al. [87] studied genomic changes and expression differences of homeologs within natural populations of *Tragopogon miscellus*, as well as in synthetic F_1 _hybrids and first-generation polyploids formed from the diploid parents *T. dubius *and *T. pratensis*. Most of the genes analyzed show additivity in *T. miscellus *at both the genomic (seven out of 23) and cDNA levels (12 out of 17). However, loss of one parental homeolog was observed at several loci (27 out of 46 homeologs), as were several examples of gene silencing (nine out of 34 homeologs). Both homeolog losses and silencing patterns vary among individuals in natural polyploid populations of independent origin [26,87]. Changes were also detected in rDNA content [71] and expression [72] in populations of *T. miscellus*. Although *T. miscellus *has fewer rDNA repeats of *T. dubius *than of *T. pratensis *[71], apparently due to concerted evolution, most of the rDNA expression derives from the *T. dubius *repeats [72]. The same pattern of rDNA expression has been observed in populations of *T. mirus *compared to its parents [71,72]; *T. mirus *has fewer repeats of *T. dubius *than of *T. porrifolius *[71], but most of the rRNA is produced by the *T. dubius *copies [72]. Although homeolog loss events and expression changes were observed in natural populations of *T. miscellus*, no such changes were observed in comparable analyses of F_1 _hybrids between the diploid parents, *T. dubius *and *T. pratensis *[26,87], or in first-generation synthetic lines [87].

In this study we extend our examination of gene loss and differential expression to the polyploid *T. mirus*. In nature, *T. mirus *has formed repeatedly, but only when *T. dubius *is the paternal parent and *T. porrifolius *is the maternal parent [69,82]. However, *T. mirus *can be produced synthetically in both directions with about equal frequency [88]. *Tragopogon mirus *provides an opportunity to compare expression differences at the genomic and transcriptional levels with the results obtained for *T. miscellus *[26,87]. Our main objectives were to: 1) investigate the genomic changes and expression differences of parental homeologs in *T. mirus *relative to its diploid parents, 2) determine the identity of the genes that exhibit those changes, and 3) assess whether individuals within and among recurrently formed natural populations of *T. mirus *show similar patterns of genome evolution and gene expression.

## Results

### cDNA-AFLP polymorphism and identification of putatively differentially expressed genes

We used cDNA-AFLPs [26,89,90]as a first step toward identifying genes with putative differential expression in the allotetraploid *T. mirus*, relative to its diploid parents (*T. dubius *and *T. porrifolius*). From our initial screen with 37 primer pairs, 1,440 fragments were produced, and of these, 504 were monomorphic (35.0%), and 936 were polymorphic (65.0%) among the three species. Novel cDNA-AFLP bands in the polyploid plants comprised 0.4% (6 fragments) of all fragments, fragments in the polyploids of maternal origin constituted 5.0% (72 fragments) of all fragments, while fragments having a paternal origin in the polyploids made up 3.5% (51 fragments) of all fragments. From this initial screening, we selected for further study 21 of the 37 primer sets, which produced an average of 50 different fragments per primer pair. From the remaining 16 primer sets, we obtained an average of 24 different fragments, but these were too short (below 250 bp) for further analysis. We then conducted an analysis on an expanded sample of the Pullman-1 population and its progenitors (10 individuals of *T. mirus*, 10 individuals of *T. porrifolius*, and 6 individuals of *T. dubius*) to obtain a larger set of potentially informative fragments. From the 21 primer pairs, 1,056 fragments were produced, and of these, 375 were monomorphic (35.5%), and 681 were polymorphic (64.5%) (Table 1). Novel cDNA-AFLP fragments in the polyploids comprised 0.6% (6 fragments) of all fragments. Shared fragments with a maternal or paternal origin in the polyploids represented 6.3% (67 fragments) or 4.6% (49 fragments) of the total fragments, respectively.

For the Pullman-2 and Palouse sites, we selected four primer sets (*Eco*RI-AA/*Mse*I-CTT, *Eco*RI-AG/*Mse*I-CTT, *Eco*RI-AG/*Mse*I-CAT, *Eco*RI-TG/*Mse*I-CTT) that showed high variation in populations from the Pullman-1 site. At the Pullman-2 site, 234 fragments were scored, and of these, 116 were monomorphic (49.6%), and 118 were polymorphic (50.4%) (Table 1). Novel cDNA-AFLP bands in the polyploids accounted for 0.4% (1 fragment) of all fragments, and fragments of maternal or paternal origin in the polyploids made up 7.7% (18 fragments) and 8.1% (19 fragments) of all bands, respectively.

At the Palouse site, 251 fragments were scored, and of these, 79 were monomorphic (31.5%), and 172 were polymorphic (68.5%) (Table 1). Fragments with a maternal or paternal origin in the polyploids made up 6.8% (17 fragments) and 5.2% (13 fragments) of all fragments, respectively. No novel cDNA-AFLP bands were detected in polyploid plants from Palouse. When we compared 20 individuals of *T. mirus *from the Pullman-1, Pullman-2, and Palouse populations, we observed very similar patterns in the Pullman-1 and Pullman-2 populations. However, individuals of *T. mirus *from Palouse have more complex patterns than individuals of *T. mirus *from Pullman-1 and Pullman-2. Individuals 2602-1 and 2602-3 from Palouse shared an AFLP pattern, whereas 2602-2 and 2602-4 showed a different pattern. There are at least three genotypes among the five individuals of *T. mirus *from Palouse.

With 125 variable fragments (>350 bp) identified from cDNA-AFLP analyses, we then searched for fragment identity based on sequence similarity using BLAST searches and identified 33 putative genes in *T. mirus *(Table 2). Further comparison with the *Arabidopsis *genome indicated that these genes are involved in various cellular processes, such as carbohydrate metabolism, signal transduction, protein transport and degradation, and cell division (Table 2). However, we could not reliably identify the remainder of the fragments because of their short length (~150 bp).

### Rapid loss of parental homeologs

The genes, enzymes, and sizes of digested genomic and cDNA amplifications for CAPS analysis of *T. mirus *and its parents are listed in Additional file 1.

**Figure 1:**
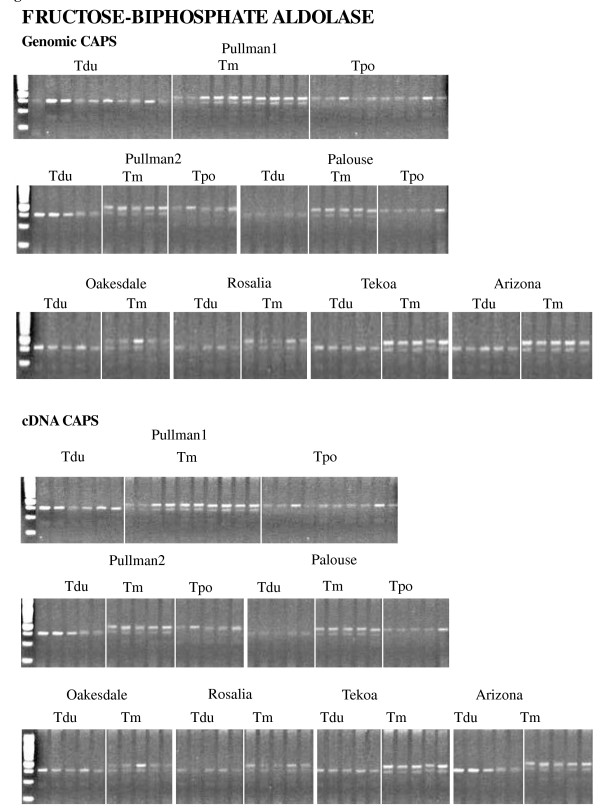
**Genomic and cDNA CAPS analyses for a putative homolog of *B-fructosidase*, an example of an additive pattern, from multiple individuals from several populations of independent origin of *T. mirus *and the parental diploids *T. dubius *and *T. porrifolius*. **Tdu = *T. dubius*, Tm = *T. mirus*, Tpo = *T. porrifolius*.

**Figure 2:**
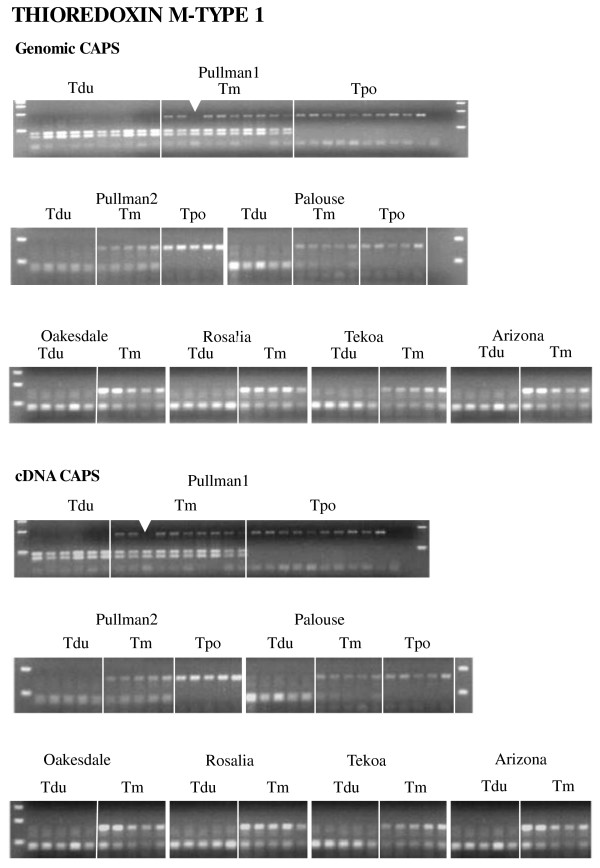
**Genomic and cDNA CAPS analyses illustrating homeolog loss in a putative homolog of *Thioredoxin M-type 1 *from multiple individuals from several populations of independent origin of *T. mirus*; also shown are the parental diploids, *T. dubius *and *T. porrifolius*.** Tdu = *T. dubius*, Tm = *T. mirus*, Tpo = *T. porrifolius*. Arrows indicate homeolog loss.

For the genomic CAPS analysis, 20 of 30 genes showed additivity, with both parental copies maintained in all allopolyploid individuals (Figure 1). Nine of 30 genes showed that at least one allotetraploid individual was missing one parental homeolog (Figure 2, Table 3). To determine whether these losses were due to true homeolog loss or simply loss of a restriction site (due to sequence polymorphism), we sequenced the PCR fragments of all genes exhibiting putative losses. Sequencing revealed that all individuals exhibiting apparent loss events have only one parental homeolog, confirming that these inferred homeolog losses are not due to restriction site divergence and loss of a CAPS marker.

**Figure 3:**
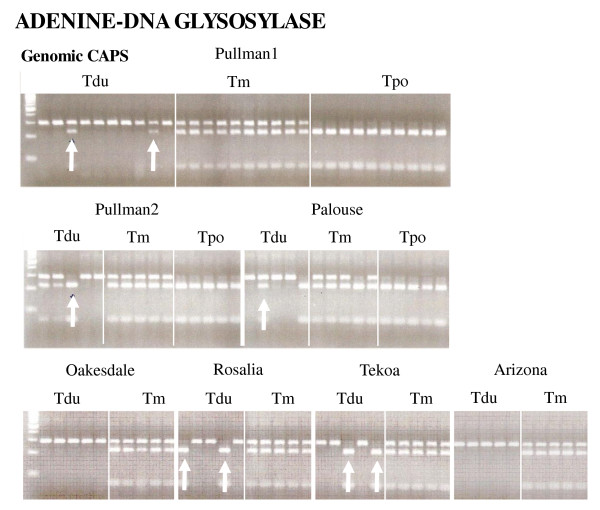
**Genomic CAPS analysis of *Adenine-DNA glycosylas*e, which exhibits a polymorphic pattern in the parental diploid *T. dubius *(see arrows)**. An additive pattern is consistently seen in the polyploid *T. mirus*. Tdu = *T. dubius*, Tm = *T. mirus*, Tpo = *T. porrifolius*.

Two genes exhibited homeolog losses of one parental copy or the other in at least one individual, whereas seven genes showed loss of only the *T. dubius *homeolog. For the putative homolog of *Thioredoxin M-type 1*, one Pullman-1 *T. mirus *plant (2601-10) showed loss of the *T. porrifolius *band, while one plant of Palouse *T. mirus *(2602-4) exhibited loss of the *T. dubius *band (Figure 2, Table 3). For the putative *Nucleic acid binding *homolog, eight *T. mirus *plants from the Pullman-2 (2678-3 and 2678-11), Oakesdale (2673-4), and Arizona (1747-1, 1747-2, 1747-3, 1747-6, 1747-9) populations showed loss of the *T. porrifolius *band, while one Palouse *T. mirus *individual (2602-25) lost the *T. dubius *band. In contrast, preferential loss of the *T. dubius *parental homeolog was observed in several individuals for seven genes (putatively identified as *Myosin heavy chain CLASS xI, LRR protein, Prenyltransferase, NADP/FAD oxidoreductase, Tetratricopeptide repeat protein, RNA binding*, and *Glyceraldehyde-3-phosphate dehydrogenase*). In addition, variation was observed among populations; losses of the *T. dubius *homeolog occurred at more loci in the Pullman-1, Pullman-2, and Palouse populations than in the Oakesdale, Rosalia, Tekoa, and Arizona populations. For example, individuals from the Palouse population showed loss of the *T. dubius *homeolog for four genes, while individuals from the Oakesdale population exhibited gene loss for only one gene (Table 3). Therefore, the Pullman-1, Pullman-2, and Palouse populations of *T. mirus *show higher levels of, and greater variation in, homeolog loss than do populations from Oakesdale, Rosalia, Tekoa, and Arizona. However, one putative gene (*Adenine-DNA glycosylase*) was polymorphic in both *T. mirus *and *T. dubius *(Figure 3). Most individuals of *T. dubius *have a single copy of this gene, but five *T. dubius *individuals have an extra copy that corresponds to the PCR amplicon produced in *T. porrifolius*. Also, six *T. dubius *individuals only have the "*T. porrifolius*" type (Figure 3). This polymorphism observed in *T. dubius *can affect interpretation of the expression patterns of *T. mirus*, making it hard to distinguish loss from polymorphism. As a result of this polymorphism, this gene was not employed in our analyses of loss events.

**Figure 4:**
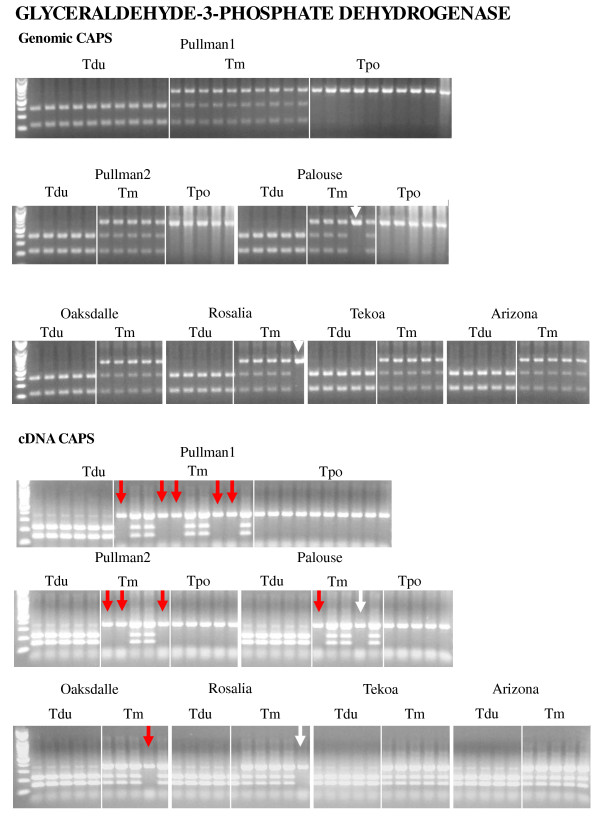
**Genomic and cDNA CAPS analyses illustrating homeolog loss as well as silencing in a putative homolog of *Glyceraldehyde-3-phosphate dehydrogenase *from multiple individuals from several populations of independent origin of *T. mirus*; also shown are the parental diploids, *T. dubius *and *T. porrifolius***. Tdu = *T. dubius*, Tm = *T. mirus*, Tpo = *T. porrifolius*. White arrows indicate homeolog loss and red arrows show silencing.

cDNA CAPS analysis was performed for 15 of 30 genes. The remaining 15 genes analyzed above for genomics CAPS could not be amplified (see Materials and Methods). Eleven of the genes included in the cDNA analyses showed additivity, whereas four genes (putative homologs of *Thioredoxin M-type 1*, *Myosin heavy chain CLASS XI*, *Nuclear ribosomal DNA*, and *Glyceraldehyde-3-phosphate dehydrogenase*) showed expression differences in some polyploid individuals relative to the diploid parents. However, the apparent expression differences from the *Thioredoxin M-type 1 *and *Myosin heavy chain CLASS XI *result from genomic losses (see above; Figure 2, Additional file 2). For the other two genes, true expression differences were detected. For the putative homolog of *Nuclear ribosomal DNA*, cDNA CAPS showed absence of the *T. dubius *homeolog in one individual of *T. mirus *(2602-3) from Palouse, while genomic CAPS found additive patterns in all tetraploid individuals (Additional file 3). Also, a putative homolog of *Glyceraldehyde-3-phosphate dehydrogenase *showed silencing in *T. mirus *in six individuals from Pullman-1 (2601-5, 2601-10, 2601-12, 2601-14, 2601-45, and 2601-47), three individuals from Pullman-2 (2678-1, 2678-2, and 2678-11), one individual from Palouse (2602-1), and one individual from Oakesdale (2673-5) (Figure 4, Table 4).

In summary, 27 of 40 individuals sampled of *T. mirus *showed loss of at least one homeolog, and 12 individuals exhibited true loss of expression of one parental homeolog (Tables 3, 4).

### Diploid F_1 _hybrids are additive of their parental genomes

Genomic CAPS analyses for six synthetic F_1 _hybrids from two independent crosses between *T. dubius *and *T. porrifolius *were also performed for the same 30 genes surveyed in natural populations of *T. mirus*. Significantly, all genomic CAPS analyses of F_1 _hybrids exhibited additivity of the parental homeologs. cDNA CAPS analysis for all 15 genes investigated in natural populations of *T. mirus *showed that both parental homeologs were expressed in all F_1 _hybrids examined (Additional file 4).

## Discussion

### cDNA-AFLP variation in populations of *T. mirus*

As cDNA-AFLPs reveal potentially differentially expressed genes, the results can provide useful initial information on the genetics of polyploids, especially those that lack developed genomic resources, such as *Tragopogon*. Thus, cDNA-AFLPs provide numerous candidate genes relatively quickly and inexpensively. However, cDNA-AFLP analysis must be followed by other approaches, such as CAPS analysis, because cDNA-AFLP fragment differences may result from true expression differences, sequence polymorphism, or gene or homeolog loss [26,91].

From cDNA-AFLPs, we identified 33 putative genes that were not additive of the parental bands in *T. mirus*; most of these (21 out of 33) exhibited maternal banding patterns (Table 2). However, subsequent analysis of 23 of these genes using genomic and cDNA CAPS analyses showed that only four of these genes exhibited an expression difference; two genes, *THIOREDOXIN M-TYPE 1 *and *MYOSIN HEAVY CHAIN CLASS XI*, showed homeolog losses, while *NUCLEAR RIBOSOMAL DNA *and *GLYCERALDEHYDE-3-PHOSPHATE DEHYDROGENASE *exhibited true silencing (Figure 4, Table 4). However, most of the genes exhibiting apparent maternal, paternal, or novel banding patterns in cDNA-AFLPs actually showed additive patterns in genomic and cDNA CAPS analyses, indicating that the cDNA-AFLP fragment differences observed in *T. mirus *may be derived from sequence polymorphism and are not indicative of homeolog loss or silencing.

### Rapid genomic changes in *T. mirus*

Analyses of CAPS markers provide evidence of rapid, frequent, and preferential elimination of homeologous loci and changes in gene expression in allotetraploid individuals of *T. mirus*. Most of the genes examined showed genomic CAPS patterns that are additive of the parental genes, but nine genes of the 30 examined showed homeolog loss, fewer than observed for *T. miscellus*, in which 16 loci of the 23 examined showed homeolog loss [26,86,87].

In *T. mirus*, the *T. dubius *parental homeolog has been lost at more loci (12.1%; 7 out of 58 homeologs) than the *T. porrifolius *homeolog (6.9%; 4 out of 58 homeologs). Likewise, in *T. miscellus*, *T. dubius *parental homeolog has been lost at more loci (32.6%; 15 out of 46 homeologs) than the *T. pratensis *homeolog (26.1%; 12 out of 46 homeologs) [26,86,87]. However, the *T. porrifolius *homeolog has actually been lost from more individuals of *T. mirus *(25) than has the *T. dubius *homeolog (10), in contrast to data for *T. miscellus *[26,87]. This difference between *T. mirus *and *T. miscellus *results from the extensive loss of the *T. porrifolius *homeolog for *NADP/FAD OXIDOREDUCTASE*. Furthermore, each homeolog absence in a polyploid should not necessary be viewed as a unique loss, as a single loss may subsequently be transmitted throughout a population.

The preferential loss of *T. dubius *homeologs also agrees with the biased rDNA homogenization of *T. dubius *repeats to the other diploid parental type in both *T. miscellus *and *T. mirus *[71]. That is, the number of *T. dubius *rDNA units has typically been greatly reduced in the genomes of the allotetraploids *T. mirus *and *T. miscellus *relative to the other diploid parent (either *T. porrifolius *or *T. pratensis*) due to apparent concerted evolution [71,72]. The single exception is the Palouse population of *T. mirus*, in which individuals have a relatively high number of *T. dubius *rDNA repeats relative to *T. porrifolius *repeats (compared to the diploid parental plants from the Palouse) [71], illustrating variation in rDNA repeat composition among populations of *T. mirus *of independent origin. In contrast, allopolyploids in *Arabidopsis *and *Brassica *apparently have not undergone loss or concerted evolution of rDNA units [92,93]. However, rapid concerted evolution of rDNA units in just a few generations [94,95] has occurred in some synthetic hybrids and allotetraploids, including synthetic hybrids between maize and *Tripsacum *[96], somatic hybrids of *Medicago sativa *[97], synthetic *Nicotiana *allotetraploids [94,98], and synthetic allotetraploid *Arabidopsis suecica *[99].

Despite being fewer in number, the rDNA units of *T. dubius *origin dominate rDNA transcription in most populations of *T. mirus *[72]. rDNA gene reduction by concerted evolution in allopolyploids can therefore be countered by high levels of expression controlled by epigenetic regulation. We obtained similar results for rDNA expression here in our cDNA CAPS study. Based on visual comparison of banding intensity, there is higher rDNA expression of *T. dubius*-origin units than of *T. porrifolius*-origin units in all plants from all populations examined, except for individuals from the Palouse population (Additional file 3). Interestingly, for the Palouse population, cDNA and genomic CAPS indicate the silencing of the *T. dubius *rDNA unit in one plant of *T. mirus*. Hence, these results further highlight the importance of populational surveys, by indicating some stochasticity for rDNA expression in the young polyploid *T. mirus*.

Some synthetic and natural allopolyploids show remarkable genomic restructuring (e.g., *Arabidopsis suecica *[56], *Brassica napus *[51,54,55], *Nicotiana *lines [94,95,100], *Primula *[101], and wheat [48]). For example, synthetic *Brassica napus *allopolyploids exhibit many chromosomal translocations and transposition events during the S_2 _to S_5 _generations, based on RFLP analysis of synthetic lines [51,55]. Genome evolution in the natural polyploids *Tragopogon mirus *and *T. miscellus *appears most similar to the results obtained for these synthetic *Brassica *allopolyploids. Homeolog loss appears frequent in both systems. Recent cytogenetic studies using FISH and GISH indicate that both *T. mirus *and *T. miscellus *show evidence of rapid genomic rearrangement, including translocations and inversions [102]. Genetic changes observed in synthetic *Brassica napus *as well as natural populations of *Tragopogon mirus *and *T. miscullus *may be related, in part, to chromosomal rearrangements in these polyploids [51,55,102].

Plants of the synthetic and naturally occurring allopolyploid *Arabidopsis suecica *also exhibit chromosomal rearrangement [56], as well as many changes in gene expression [40,41]. Genes from one parent, *A. thaliana*, have often been silenced epigenetically by DNA methylation [41]. Through microarray analysis, Wang et al. [40] showed that approximately 65% of nonadditively expressed genes in the synthetic allotetraploids were repressed, and more than 94% of them matched the genes that are highly expressed in one parent, *A. thaliana*. *Tragopogon *allopolyploids have undergone many losses of homeologous loci, often eliminating copies of one parent, *T. dubius*, but fewer instances of gene silencing. Additional studies of *Tragopogon *are needed to similarly examine gene expression on a genomic-level scale.

F_1 _diploid hybrids and early synthetic allotetraploids (S_1 _to S_3 _generation) between *Aegilops sharonensis *and *Triticum monococcum *ssp. *aegilopoides *showed both gene loss and silencing by DNA methylation [1,47,48,103-105]. However, such immediate changes have not been detected in *Tragopogon*. Genomic and cDNA CAPS data for synthetic F_1 _hybrids between *T. dubius *and *T. porrifolius *showed additivity rather than gene loss or silencing (Additional file 4). Similarly, no homeolog loss was observed in synthetic F_1 _hybrids between *T. dubius *and *T. pratensis *[26], or in newly produced (S_0_) or first-generation (S_1_) synthetic *T. miscellus *[87]. Therefore, in contrast to wheat, both *T. mirus *and *T. miscellus *exhibit genome evolution, not immediately following hybridization or allopolyploidization, but apparently shortly thereafter, given that the species are probably less than 80 years old (or 40 generations; these plants are biennials) [69,70]. Also, these results suggest that loss of homeologs and gene expression changes, while still rapid in evolutionary time, may be slightly more gradual in *Tragopogon*, occurring over several generations, but further studies are required to assess the speed and magnitude with which genomic changes have occurred in *Tragopogon*.

A major question centers on the mechanisms responsible for the loss of homeologs in these young allopolyploids. Ownbey [69] observed the formation of complex multivalents during meiosis in both *T. mirus *and *T. miscellus *shortly after their formation and in F_1 _hybrids between *T. dubius *and *T. porrifolius*, and between *T. dubius *and *T. pratensis*. Furthermore, multivalents have also been observed in synthetic *T. mirus *and *T. miscellus *[88,106]. In addition, rare patterns observed in analysis of allozyme variation in *Tragopogon *are consistent with non-homologous recombination [84]. Non-homologous recombination could provide a mechanism of homeolog loss in *T. mirus *and *T. miscellus*, as in *Brassica *[55]. Recent cytogenetic data provide additional insights into potential mechanisms for gene loss in *Tragopogon*. GISH studies have revealed that chromosomal rearrangements and other changes may be common in natural populations of *T. mirus *and *T. miscellus *[102]. Intergenomic translocations, inversions, as well as apparent monosomy and reciprocal trisomy occur in fertile individuals of both polyploids [102]. Such rearrangements provide a potential mechanism for the homeolog losses observed in both *T. mirus *and *T. miscellus*.

### Genomic changes versus differential expression in *T. mirus*

For most of the genes examined here, homeolog losses appear to be responsible for the cDNA-AFLP fragment differences observed in individuals of *T. mirus *relative to its diploid progenitors. However, in two genes (putatively *Nuclear ribosomal DNA *and *Glyceraldehyde-3-phosphate dehydrogenase*), we found true expression differences in the allopolyploid relative to its parents. *Nuclear ribosomal DNA *encodes ribosomal RNA, and *Glyceraldehyde-3-phosphate dehydrogenase *encodes an enzyme that participates in multiple processes, including transcription activation, initiation of apoptosis, and ER to Golgi vesicle shuttling [107], so both of these genes are crucial for cell function. The pattern of *Nuclear ribosomal DNA *from genomic and cDNA CAPS analyses is consistent with Matyášek et al.'s [72] study: all individuals from the Palouse population showed additivity with genomic CAPS and in the Southern blot rDNA study of Matyášek et al. [72]. However, in one individual from the Palouse population, the *T. dubius Nuclear ribosomal DNA *homeolog was completely silenced in both our cDNA CAPS analysis and in the rDNA transcript study of Matyášek et al. [72].

Most of the *T. mirus *individuals examined here, except those from Tekoa and Arizona, show no expression of the *T. dubius *homeolog for *Glyceraldehyde-3-phosphate dehydrogenase *(Figure 4). Genomic CAPS data indicate homeolog loss in two individuals, but additivity of parental homeologs in the remaining individuals. cDNA CAPS analyses therefore exhibit gene silencing in 13 individuals (Figure 4). Recent studies have shown that rapid epigenetic gene silencing following allopolypoid formation can be reversed by chemical demethylation in allopolyploid *Arabidopsis suecica *[41,43,44,108]. Therefore, silencing of these two genes might result from epigenetic phenomena such as DNA methylation or histone acetylation [109,110].

In this study, gene silencing of *T. dubius *homeologs occurred in only two cases. In addition, expression studies of *T. miscellus *[26,87] showed that seven out of 17 genes exhibited silencing of the *T. dubius *homeolog, while for two other genes, the *T. pratensis *homeolog was silenced. These biased patterns in *T. mirus *and *T. miscellus *indicate that *T. dubius *homeologs might be more susceptible to silencing than the alternative parental homeologs.

When we compare gene silencing with gene loss with respect to the number of individuals examined, the previous studies of *T. miscellus *[26,87] show that silencing events are slightly more frequent than homeolog loss, while in this study of *T. mirus*, homeolog losses are slightly more frequent than silencing events. These expression patterns result from biased expression of only a few genes. For example, 14 out of 20 *T. miscellus *individuals have silencing events in *Leucine-rich repeat transmembrane protein kinase*, and 11 out of 40 *T. mirus *individuals have silencing events in *glyceraldehyde-3-phosphate dehydrogenase*. Therefore, comparing gene loss with silencing events in *T. mirus *and *T. miscellus *seems to be affected by specific genes, with a few of the genes examined here especially prone to silencing.

However, when we consider the number of genes examined in this study of *T. mirus *and in the previous *T. miscellus *studies [26,86,87], homeolog losses in *T. mirus *(18.97%; 11 out of 58 homeologs) and in *T. miscellus *(58.7%; 27 out of 46 homeologs) are more frequent than silencing events (6.7%; two out of 30 homeologs in *T. mirus*; 26.4%; nine out of 34 homeologs in *T. miscellus*). Nevertheless, we investigated only a small portion of the genome, so further studies are required to assess whether the results for the ~30 genes so far examined are representative of the entire genome.

Genomic CAPS analysis of a putative *Adenine DNA glycosylase *gene showed polymorphism in the populations of the diploid parent *T. dubius *surveyed here (Figure 3). Although previous studies in *Tragopogon *diploids using allozymes and other markers indicated that genetic variation within populations is quite low [83,84], *T. dubius *is the most genetically variable of the three diploids; allozyme variation within populations of *T. porrifolius *and *T. pratensis *was limited or absent. Therefore, polymorphism among *T. dubius *individuals for one of the genes analyzed here is not surprising. A recent survey of *Tragopogon *diploids and polyploids from the Palouse using microsatellite markers similarly revealed low levels of genetic variation within populations of *T. dubius*, but none within either *T. pratensis *or *T. porrifolius *(V. Symonds et al., unpublished data).

## Conclusions

Recently formed *Tragopogon *allotetraploids (<80 years; 40 generations for these biennial plants) exhibit various consequences of genome evolution and gene expression following polyploidy. In this study, using cDNA-AFLPs, we found differential banding patterns, possibly attributable to gene silencing, novel expression, and/or maternal/paternal effects between *T. mirus *and its diploid parents. Most of the banding patterns subsequently investigated with genomic and cDNA CAPS analyses revealed additivity. Most of the differences observed in *T. mirus *result from homeolog loss, rather than gene silencing; the latter was detected only infrequently (in two genes in some individuals). Genomic and cDNA CAPS analyses indicated that plants of *T. mirus *have experienced frequent and preferential elimination of the *T. dubius *homeolog, whereas comparable analyses of synthetic F_1 _hybrids between the parents (*T. dubius *× *T. porrifolius*) of *T. mirus *showed only additivity.

These same results were also obtained for the recently and repeatedly formed allotetraploid *Tragopogon miscellus *[26,87]. Both *T. mirus *and *T. miscellus *undergo biased loss of homeologs contributed by their shared diploid parent, *T. dubius*. Furthermore, both allotetraploids exhibit more homeolog losses than gene silencing in terms of the number of genes undergoing change. Taken together, our results suggest that in *Tragopogon *loss of homeologs and gene silencing are not immediate consequences of hybridization or polyploidization, but are processes that occur following polyploidization, occurring over a relatively small number of generations. These results further support the idea of polyploidy as a dynamic evolutionary process (reviewed in 117), with abundant and rapid genomic changes occurring within a short time period following polyploidization. Further studies of homeolog loss, nonadditive expression patterns, and subfunctionalization of homeologs are needed to explore the roles of genetic and epigenetic phenomena in the evolution of allotetraploid *Tragopogon *species.

## Methods

### Plant materials

For populations Pullman-1 and -2, Palouse, and Rosalia, seeds were collected from natural populations and grown in the greenhouse at Washington State University (Pullman, WA, USA) and allowed to self-fertilize. Seeds from these greenhouse-grown plants were collected, germinated, and grown under controlled conditions in the greenhouse at the University of Florida (UF; Gainesville, FL, USA). Seeds from the Oakesdale, Tekoa, and Arizona populations were collected and then grown at UF without a round of selfing. Each population of *T. mirus *is inferred to be of separate origin (V. Symonds et al., unpublished data) and was analyzed, along with samples of the diploid progenitors from each location (Table 5; [70,83,84]). However, only the paternal parent, *T. dubius*, was investigated for populations from Oakesdale, Rosalia, Tekoa, and Arizona because *T. porrifolius *was not found at those sites.

Diploid F_1 _hybrids used in this study were generated by J. Tate, who crossed *T. dubius *(2611-11, Pullman-1; the paternal progenitor) and *T. porrifolius *(2613-24, Pullman-1; the maternal progenitor) using plants grown from seed in the greenhouse[88].

### cDNA-AFLP display and identification of polymorphic fragments

Here, following Tate et al. [26], we initially employed cDNA-amplified fragment length polymorphisms (cDNA-AFLPs) to identify potentially differentially expressed genes [111]. This approach has proven to be useful in systems without well-developed genetic resources [1,26,43,91,112-114]. However, the weakness of this approach is that fragment differences observed on a cDNA-AFLP gel may result from true expression differences, sequence polymorphisms, or gene or homeolog loss [26,40]. Due to this limitation, we subsequently examined the expression patterns of genes isolated from cDNA-AFLPs using genomic and cDNA CAPS analysis [26,115]. This approach can determine whether apparent expression differences observed at the transcriptional level result from genomic changes such as gene loss or from true differences in expression. Putative homeolog losses were further tested via DNA sequencing.

Leaf segments less than 30 cm in length were collected from young plants six weeks after germination. Due to heavy latex in the leaf tissue, we extracted total RNA from leaf tissue using the method of Kim et al. [116], which combines a CTAB extraction protocol [117] and subsequent use of the RNeasy Plant Mini Kit (Qiagen, Valencia, CA, USA). cDNA was synthesized from total RNA using SuperScript Double-Stranded cDNA Synthesis Kit (Invitrogen, Carlsbad, CA, USA), and the cDNA-AFLP technique was performed as previously described [43], except that we replaced isotope-based signal detection with silver staining [118].

To investigate the utility of cDNA-AFLPs in *T. mirus*, we conducted an initial survey of the Pullman-1 population with 37 primer combinations. Following the success of this survey, expanded cDNA-AFLP analyses were conducted on 5-10 individuals each from the Pullman-1, Pullman-2, and Palouse populations of *T. mirus *and from the diploid progenitors that occurred with the tetraploid populations. We employed the same methods, using the primer sets that were the most variable in our initial screen (21 for Pullman-1 and four each for Pullman-2 and Palouse; Additional file 5). We analyzed 56 individuals: 20 of *T. mirus*, 16 of *T. dubius*, and 20 of *T. porrifolius *from the Pullman-1, Pullman-2, and Palouse populations, respectively (Table 5). The expressed bands on cDNA-AFLP gels were scored as monomorphic (present in all individuals) or polymorphic (present in at least one individual/absent in at least one individual) (Table 1).

From the expanded cDNA-AFLP work, 125 variable fragments exhibiting novel, maternal/paternal, or other polymorphic patterns were identified from the Pullman-1, Pullman-2, and Palouse populations. To determine the putative identity of these fragments, we excised and sequenced fragments over 250 bp in size, as described in Lee and Chen [43], Wang et al. [91], and Tate et al. [26]. The polymorphic bands were cut from the polyacrylamide gels, and the fragments were re-amplified using the same set of selective amplification primers and cloned using a Topo TA Cloning Kit (Invitrogen). Sequencing was performed with the CEQ DTCS-Quick Start Kit (Beckman Coulter, Fullerton, CA, USA). To identify the sequences obtained above, we used BLAST searches against the NCBI database http://www.ncbi.nlm.nih.gov, and the sequence identity was rechecked against the Compositae Genome Project database http://compgenomics.ucdavis.edu. Identified sequences have been deposited in the EMBL/GenBank database under accession nos (Additional file 6).

### CAPS analyses

To determine if cDNA-AFLP fragment polymorphisms resulted from genomic changes or expression differences, we conducted both genomic and cDNA CAPS analyses. In CAPS analyses, amplified PCR products are digested with diagnostic restriction enzymes that distinguish the diploid parental sequences, and the fragments are separated by agarose gel electrophoresis.

For this study we included populations of *T. dubius *and *T. mirus *from Oakesdale, Tekoa, and Rosalia, Washington, and from Arizona, in addition to the Pullman-1, Pullman-2, and Palouse populations. Therefore, 100 individuals were examined for genomic CAPS analyses (Table 5): 40 of *T. mirus*, 40 of *T. dubius*, and 20 of *T. porrifolius*, with 5-10 individuals per population. For cDNA CAPS analyses, we analyzed 40 individuals of *T. mirus*, 36 of *T. dubius*, and 20 of *T. porrifolius*, with 5-10 individuals per population (Table 5). We also analyzed 6 F_1 _hybrid plants using both genomic and cDNA CAPS to determine whether genomic changes and expression differences appear in the first-generation hybrids.

For CAPS analyses, we used 30 primer sets: 23 primer sets were designed based on the sequences that were variable in the cDNA-AFLP analysis of the Pullman-1, Pullman-2, and Palouse populations, and 7 primer sets were from studies of *T. miscellus *[26,87]. To design primer sets, we first BLASTed our fragments against *Lactuca *or *Helianthus *ESTs and then used the "hit" ESTs for primer design because the ESTs are likely longer than the isolated fragments, with a greater chance that the expressed sequence spans the introns in genomic DNA. With those primer sets, we amplified fragments from *T. dubius *and *T. porrifolius *and then confirmed their sequences. From those sequences, we redesigned the primer sets to be more specific for analysis of *Tragopogon *CAPS. All primers were designed using the web interface program, Primer3 (v. 0.4.0; http://frodo.wi.mit.edu/). Primer sequence information is given in Additional file 6.

For each of the 30 gene regions, we aligned DNA sequences from the diploid parents using Sequencher v. 4.1.4 (Gene Codes Corporation, Ann Arbor, MI, USA) and identified diagnostic restriction sites between the species.

#### Genomic CAPS analyses

We isolated genomic DNA from 100 individuals of the allotetraploid and its two progenitors using a modified CTAB protocol [117]. Genomic fragments were amplified in a 25 μl volume with 20 ng template, 5× buffer, 1.5 mM MgCl_2_, 0.2 mM dNTPs, 0.1 mM each primer, and 0.5 unit Taq polymerase (Promega, Madison, WI, USA). Thermal cycling conditions were as follows: 95°C for 2 min, followed by 35 cycles of 95°C for 30 sec, 54-56°C for 30 sec, 72°C for 1 min 20 sec, and a final 7-min extension at 72°C. Products were separated on a 1.5% agarose gel, stained with ethidium bromide, and visualized using a UV transilluminator. Genomic digests were performed in a 12 μl volume, containing 1× buffer, 3 μl PCR product, 2 units of restriction enzyme (New England Biolabs, Ipswich, MA, USA), and 100 μg/ml bovine serum albumin (when required), and incubated at the appropriate temperature for 9 hr. Digested products were separated on 2-4% agarose gels, stained with ethidium bromide or SyberGold (Invitrogen), and visualized using a transilluminator.

To determine whether putative homeolog losses observed in genomic CAPS analyses were due to true homeolog loss or simply a loss of a restriction site (due to sequence polymorphism in one parental fragment in the polyploid), we sequenced the initial PCR product. A homeolog loss would yield only one parental diploid sequence, whereas loss of a restriction site in one parent would still yield both parental DNA sequences.

#### cDNA CAPS analyses

We isolated total RNAs from 96 of the individuals analyzed for genomic CAPS (see above) using the method of Kim et al. [116] and the RNeasy Plant Mini Kit (Qiagen). The first-strand cDNA was synthesized with 5 μg total RNA using Superscript II reverse transcriptase (Invitrogen) with a poly-T (T17) primer. Using the same primer sets as for 15 of the loci in the genomic CAPS analyses, RT-PCR was carried out using 50 ng of template from the first-strand cDNA in a 25 μl volume with 20 ng template, 5× buffer, 1.5 mM MgCl_2_, 0.2 mM dNTPs, 0.1 mM each primer, and 0.5 unit *Taq *polymerase (Promega). The remaining 15 genes could not be amplified from these cDNAs; the reasons for this are unclear, but perhaps these genes were not expressed in the leaf tissue sampled for cDNA CAPS. For amplification of fragments and digestion of RT-PCR products, we employed basically the same approach as described for the genomic CAPS analyses. In addition, for *NUCLEAR RIBOSOMAL DNA*, the relative PCR band intensities of the two homeologs were measured using KODAK 1D Image Analysis Software (Kodak, Rochester, NY, USA).

## List of abbreviations

CAPS: cleaved amplified polymorphic sequence; cDNA-AFLPs: cDNA-amplified fragment length polymorphisms.

Organisms: *T. dubius*: *Tragopogon dubius*; *T. mirus*: *Tragopogon mirus*; *T. miscellus*: *Tragopogon miscellus*; *T. porrifolius*: *Tragopogon porrifolius*; *T. pratensis*: *Tragopogon pratensis*.

## Authors' contributions

JK carried out all experiments described above, and with PSS and DES wrote the manuscript. PSS and DES designed and supervised the project. All authors read and approved the final submission.

## Supplementary Material

Additional file 1 **Supplementary Data**. Homeologous loci and restriction enzymes examined in *T. mirus *with genomic and cDNA CAPS analyses. Click here for file

Additional file 2 **Supplementary Data**. Genomic and cDNA CAPS analyses illustrating homeolog loss in a putative homolog of *MYOSIN HEAVY CHAIN CLASS XI *from multiple individuals from several populations of independent origin of *T. mirus*; also shown are the parental diploids, *T. dubius *and *T. porrifolius*. Tdu = *T. dubius*, Tm = *T. mirus*, Tpo = *T. porrifolius*. Arrows indicate missing homeologs. Click here for file

Additional file 3 **Supplementary Data**. Genomic and cDNA CAPS analyses of *NUCLEAR RIBOSOMAL DNA*, which exhibits silencing pattern in one plant of *T. mirus *from Tekoa (see red arrow). This plant is not expressing the homeolog of *T. dubius*. Tdu = *T. dubius*, Tm = *T. mirus*, Tpo = *T. porrifolius*. Click here for file

Additional file 4 **Supplementary Data**. Genomic and cDNA CAPS analyses for 15 candidate genes from *Tragopogon *F_1 _hybrids and their porgenitors. Tdu = *T. dubius*(2611-11, Pullman, WA), Tpo = *T. porrifolius *(2613-24, Pullman, WA). Click here for file

Additional file 5 **Supplementary Data**. Primer combination for selective amplification and used in cDNA-AFLP analyses. Asterisk indicates primers used in expanded study. Click here for file

Additional file 6 **Supplementary Data**. Primer. Click here for file

## References

[B1] Kashkush K Feldman M Levy AA Gene loss, silencing and activation in a newly synthesized wheat allotetraploid Genetics 2002 160 4 1651 1659 11973318 10.1093/genetics/160.4.1651 PMC1462064

[B2] Wendel J Doyle J Henry RJ Polyploidy and evolution in plants Plant diversity and evolution: genotypic and pheontypic variation in higher plants 2005 Oxfordshire: CABI 97 117 full_text

[B3] Adams KL Evolution of duplicate gene expression in polyploid and hybrid plants J Hered 2007 98 2 136 141 10.1093/jhered/esl061 17208934

[B4] Soltis DE Soltis PS Polyploidy: recurrent formation and genome evolution Trends Ecol Evol 1999 14 9 348 352 10.1016/S0169-5347(99)01638-9 10441308

[B5] Otto SP Whitton J Polyploid incidence and evolution Annu Rev Genet 2000 34 401 437 10.1146/annurev.genet.34.1.401 11092833

[B6] Goldblatt P Lewis WH Polyploidy in angiosperms: Monocotyledons Polyploidy: biological relevance 1980 New York: Plenum Press 219 239

[B7] Stebbins GL Variation and evolution in plants 1950 New York: Columbia University Press

[B8] Soltis DE Soltis PS Tate JA Advances in the study of polyploidy since plant speciation New Phytol 2003 161 173 191 10.1046/j.1469-8137.2003.00948.x

[B9] Masterson J Stomatal size in fossil plants: evidence for polyploidy in majority of angiosperms Science 1994 264 421 423 10.1126/science.264.5157.421 17836906

[B10] Zhang L Vision TJ Gaut BS Patterns of nucleotide substitution among simultaneously duplicated gene pairs in *Arabidopsis thaliana* Mol Biol Evol 2002 19 9 1464 1473 12200474 10.1093/oxfordjournals.molbev.a004209

[B11] Blanc G Hokamp K Wolfe KH A recent polyploidy superimposed on older large-scale duplications in the *Arabidopsis *genome Genome Res 2003 13 2 137 144 10.1101/gr.751803 12566392 PMC420368

[B12] Bowers JE Chapman BA Rong J Paterson AH Unravelling angiosperm genome evolution by phylogenetic analysis of chromosomal duplication events Nature 2003 422 6930 433 438 10.1038/nature01521 12660784

[B13] Zhang J Evolution by gene duplication: an update Trends Ecol Evol 2003 18 292 298 10.1016/S0169-5347(03)00033-8

[B14] Sterck L Rombauts S Vandepoele K Rouze P Peer Y Van de How many genes are there in plants (... and why are they there)? Curr Opin Plant Biol 2007 10 2 199 203 10.1016/j.pbi.2007.01.004 17289424

[B15] Arabidopsis Genome Initiative Analysis of the genome sequence of the flowering plant *Arabidopsis thaliana* Nature 2000 408 6814 796 815 10.1038/35048692 11130711

[B16] Vision TJ Brown DG Tanksley SD The origins of genomic duplications in *Arabidopsis* Science 2000 290 5499 2114 2117 10.1126/science.290.5499.2114 11118139

[B17] Simillion C Vandepoele K Van Montagu MC Zabeau M Peer Y Van de The hidden duplication past of *Arabidopsis thaliana* Proc Natl Acad Sci USA 2002 99 21 13627 13632 10.1073/pnas.212522399 12374856 PMC129725

[B18] Tuskan GA Difazio S Jansson S Bohlmann J Grigoriev I Hellsten U Putnam N Ralph S Rombauts S Salamov A The genome of black cottonwood, *Populus trichocarpa *(Torr. & Gray) Science 2006 313 5793 1596 1604 10.1126/science.1128691 16973872

[B19] Velasco R Zharkikh A Troggio M Cartwright DA Cestaro A Pruss D Pindo M Fitzgerald LM Vezzulli S Reid J A high quality draft consensus sequence of the genome of a heterozygous grapevine variety PLoS ONE 2007 2 12 e1326 10.1371/journal.pone.0001326 18094749 PMC2147077

[B20] Jaillon O Aury JM Noel B Policriti A Clepet C Casagrande A Choisne N Aubourg S Vitulo N Jubin C The grapevine genome sequence suggests ancestral hexaploidization in major angiosperm phyla Nature 2007 449 7161 463 467 10.1038/nature06148 17721507

[B21] Ku HM Vision T Liu J Tanksley SD Comparing sequenced segments of the tomato and Arabidopsis genomes: large-scale duplication followed by selective gene loss creates a network of synteny Proc Natl Acad Sci USA 2000 97 16 9121 9126 10.1073/pnas.160271297 10908680 PMC16832

[B22] Cui L Wall PK Leebens-Mack JH Lindsay BG Soltis DE Doyle JJ Soltis PS Carlson JE Arumuganathan K Barakat A Widespread genome duplications throughout the history of flowering plants Genome Res 2006 16 6 738 749 10.1101/gr.4825606 16702410 PMC1479859

[B23] Scannell DR Byrne KP Gordon JL Wong S Wolfe KH Multiple rounds of speciation associated with reciprocal gene loss in polyploid yeasts Nature 2006 440 7082 341 345 10.1038/nature04562 16541074

[B24] Lynch M Conery JS The evolutionary fate and consequences of duplicate genes Science 2000 290 1151 1155 10.1126/science.290.5494.1151 11073452

[B25] Prince VE Pickett FB Splitting pairs: the diverging fates of duplicated genes Nat Rev Genet 2002 3 11 827 837 10.1038/nrg928 12415313

[B26] Tate JA Ni Z Scheen AC Koh J Gilbert CA Lefkowitz D Chen ZJ Soltis PS Soltis DE Evolution and expression of homeologous loci in *Tragopogon miscellus *(Asteraceae), a recent and reciprocally formed allopolyploid Genetics 2006 173 3 1599 1611 10.1534/genetics.106.057646 16648586 PMC1526671

[B27] Force A Lynch M Pickett FB Amores A Yan YL Postlethwait J Preservation of duplicate genes by complementary, degenerative mutations Genetics 1999 151 4 1531 1545 10101175 10.1093/genetics/151.4.1531 PMC1460548

[B28] Lynch M Conery JS The evolutionary fate of duplicated genes Science 2000 290 1151 1154 10.1126/science.290.5494.1151 11073452

[B29] Lynch M Force A The probability of duplicate gene preservation by subfunctionalization Genetics 2000 154 1 459 473 10629003 10.1093/genetics/154.1.459 PMC1460895

[B30] Lynch M O'Hely M Walsh B Force A The probability of preservation of a newly arisen gene duplicate Genetics 2001 159 4 1789 1804 11779815 10.1093/genetics/159.4.1789 PMC1461922

[B31] Adams KL Cronn R Percifield R Wendel JF Genes duplicated by polyploidy show unequal contributions to the transcriptome and organ-specific reciprocal silencing Proc Natl Acad Sci USA 2003 100 8 4649 4654 10.1073/pnas.0630618100 12665616 PMC153610

[B32] Benderoth M Textor S Windsor AJ Mitchell-Olds T Gershenzon J Kroymann J Positive selection driving diversification in plant secondary metabolism Proc Natl Acad Sci USA 2006 103 24 9118 9123 10.1073/pnas.0601738103 16754868 PMC1482576

[B33] Drea SC Lao NT Wolfe KH Kavanagh TA Gene duplication, exon gain and neofunctionalization of OEP16-related genes in land plants Plant J 2006 46 5 723 735 10.1111/j.1365-313X.2006.02741.x 16709189

[B34] Teshima KM Innan H Neofunctionalization of duplicated genes under the pressure of gene conversion Genetics 2008 178 3 1385 1398 10.1534/genetics.107.082933 18245342 PMC2278071

[B35] Tirosh I Barkai N Comparative analysis indicates regulatory neofunctionalization of yeast duplicates Genome Biol 2007 8 4 R50 10.1186/gb-2007-8-4-r50 17411427 PMC1895995

[B36] Van Damme EJ Culerrier R Barre A Alvarez R Rouge P Peumans WJ A novel family of lectins evolutionarily related to class V chitinases: an example of neofunctionalization in legumes Plant Physiol 2007 144 2 662 672 10.1104/pp.106.087981 17098856 PMC1914163

[B37] Ohno S Evolution by Gene Duplication 1970 Germany/New York: Springer-Verlag, Berlin/Heidelberg

[B38] Des Marais DL Rausher MD Escape from adaptive conflict after duplication in an anthocyanin pathway gene Nature 2008 454 7205 762 765 18594508 10.1038/nature07092

[B39] Madlung A Tyagi AP Watson B Jiang H Kagochi T Doerge RW Martienssen R Comai L Genomic changes in synthetic *Arabidopsis *polyploids Plant J 2005 41 2 221 230 10.1111/j.1365-313X.2004.02297.x 15634199

[B40] Wang J Tian L Lee HS Wei NE Jiang H Watson B Madlung A Osborn TC Doerge RW Comai L Genomewide nonadditive gene regulation in *Arabidopsis *allotetraploids Genetics 2006 172 1 507 517 10.1534/genetics.105.047894 16172500 PMC1456178

[B41] Wang J Tian L Madlung A Lee HS Chen M Lee JJ Watson B Kagochi T Comai L Chen ZJ Stochastic and epigenetic changes of gene expression in *Arabidopsis *polyploids Genetics 2004 167 4 1961 1973 10.1534/genetics.104.027896 15342533 PMC1471021

[B42] Lawrence RJ Earley K Pontes O Silva M Chen ZJ Neves N Viegas W Pikaard CS A concerted DNA methylation/histone methylation switch regulates rRNA gene dosage control and nucleolar dominance Mol Cell 2004 13 4 599 609 10.1016/S1097-2765(04)00064-4 14992728

[B43] Lee HS Chen ZJ Protein-coding genes are epigenetically regulated in *Arabidopsis *polyploids Proc Natl Acad Sci USA 2001 98 12 6753 6758 10.1073/pnas.121064698 11371624 PMC34425

[B44] Comai L Tyagi AP Winter K Holmes-Davis R Reynolds SH Stevens Y Byers B Phenotypic instability and rapid gene silencing in newly formed *Arabidopsis *allotetraploids Plant Cell 2000 12 9 1551 1568 10.1105/tpc.12.9.1551 11006331 PMC149069

[B45] Liu B Brubaker CL Mergeai G Cron RC Wendel JF Polyploid formation in cotton is not accompanied by rapid genomic changes Genome 2001 44 321 330 10.1139/gen-44-3-321 11444689

[B46] Zhao X-P Si Y Hanson RE Crane CF J PH Stelly DM Wendel JF Paterson AH Dispersed repetitive DNA has spread to new genomes since polyploid formation in cotton Genome Res 1998 8 479 492 9582192 10.1101/gr.8.5.479

[B47] Feldman M Liu B Segal G Abbo S Levy AA Vega JM Rapid elimination of low-copy DNA sequences in polyploid wheat: a possible mechanism for differentiation of homoeologous chromosomes Genetics 1997 147 3 1381 1387 9383078 10.1093/genetics/147.3.1381 PMC1208259

[B48] Ozkan H Levy AA Feldman M Allopolyploidy-induced rapid genome evolution in the wheat (*Aegilops*-*Triticum*) group Plant Cell 2001 13 8 1735 1747 10.1105/tpc.13.8.1735 11487689 PMC139130

[B49] Shaked H Kashkush K Ozkan H Feldman M Levy AA Sequence elimination and cytosine methylation are rapid and reproducible responses of the genome to wide hybridization and allopolyploidy in wheat Plant Cell 2001 13 8 1749 1759 10.1105/tpc.13.8.1749 11487690 PMC139131

[B50] Kashkush K Feldman M Levy AA Transcriptional activation of retrotransposons alters the expression of adjacent genes in wheat Nat Genet 2003 33 1 102 106 10.1038/ng1063 12483211

[B51] Song K Lu P Tang K Osborn TC Rapid genome change in synthetic polyploids of *Brassica *and its implications for polyploid evolution Proc Natl Acad Sci USA 1995 92 17 7719 7723 10.1073/pnas.92.17.7719 7644483 PMC41217

[B52] Lukens L Quijada P Udall J Pires JC E SM Osborn TC Genome redundancy and plasticity within ancient and recent *Brassica *crop species Biol J Linn Soc Lond 2004 82 665 674 10.1111/j.1095-8312.2004.00352.x

[B53] Lukens LN Pires JC Leon E Vogelzang R Oslach L Osborn T Patterns of sequence loss and cytosine methylation within a population of newly resynthesized *Brassica napus *allopolyploids Plant Physiol 2006 140 1 336 348 10.1104/pp.105.066308 16377753 PMC1326055

[B54] Pires JC Zhao JW Schranz ME Leon EJ Quijada PA Lukens LN Osborn TC Flowering time divergence and genomic rearrangements in resynthesized *Brassica *polyploids (Brassicaceae) Biol J Linn Soc Lond 2004 82 675 688 10.1111/j.1095-8312.2004.00350.x

[B55] Gaeta RT Pires JC Iniguez-Luy F Leon E Osborn TC Genomic changes in resynthesized *Brassica napus *and their effect on gene expression and phenotype Plant Cell 2007 19 11 3403 3417 10.1105/tpc.107.054346 18024568 PMC2174891

[B56] Pontes O Neves N Silva M Lewis MS Madlung A Comai L Viegas W Pikaard CS Chromosomal locus rearrangements are a rapid response to formation of the allotetraploid *Arabidopsis suecica *genome Proc Natl Acad Sci USA 2007 101 52 18240 18245 10.1073/pnas.0407258102 PMC539792 15604143

[B57] Urbanska KM Hurka H Landolt E Neuffer B Mummenhoff K Hybridization and evolution in *Cardamine *(Brassicaceae) at Urnerboden, Central Switzerland: biosystematic and molecular evidence Plant Syst Evol 1997 204 233 256 10.1007/BF00989208

[B58] Rosser EM A new British species of *Senecio* Watsonia 1955 3 228 232

[B59] Ashton PA Abbott RJ Multiple origins and genetic diversity in the newly arisen allopolyploid species, *Senecio cambrensis *Rosser (Compositae) Heredity 1992 68 25 32 10.1038/hdy.1992.3

[B60] Abbott RJ Lowe AJ Origins, establishment and evolution of new polyploid species: *Senecio cambrensis *and *S. eboracensis *in the British Isles Biol J Linn Soc Lond 2004 82 467 474 10.1111/j.1095-8312.2004.00333.x

[B61] Abbott RJ Ireland HE Rogers HJ Population decline despite high genetic diversity in the new allopolyploid species *Senecio cambrensis *(Asteraceae) Mol Ecol 2007 16 5 1023 1033 10.1111/j.1365-294X.2007.03169.x 17305858

[B62] Hegarty MJ Barker GL Wilson ID Abbott RJ Edwards KJ Hiscock SJ Transcriptome shock after interspecific hybridization in senecio is ameliorated by genome duplication Curr Biol 2006 16 16 1652 1659 10.1016/j.cub.2006.06.071 16920628

[B63] Hegarty MJ Hiscock SJ Genomic clues to the evolutionary success of polyploid plants Curr Biol 2008 18 10 R435 444 10.1016/j.cub.2008.03.043 18492478

[B64] Huskins CL The origin of *Spartina *× *townsendii* Genetica 1930 12 531 538 10.1007/BF01487665

[B65] Salmon A Ainouche ML Wendel JF Genetic and epigenetic consequences of recent hybridization and polyploidy in *Spartina *(Poaceae) Mol Ecol 2005 14 4 1163 1175 10.1111/j.1365-294X.2005.02488.x 15773943

[B66] Ainouche ML Baumel A Salmon A Yannic G Hybridisation, polyploidy and speciation in *Spartina *Schreb. (Poaceae) New Phytol 2004 161 165 172 10.1046/j.1469-8137.2003.00926.x

[B67] Raybould AF Gray AJ Hornby DD Collins M, Ansell K Evolution and current status of the salt marshes grass, *Spartina anglica *in the Solent Solent science - a review 2000 Amsterdam: Elsevier Science 299 302

[B68] Hubbard JCE Grimes BH Marchant CJ Some observations on the ecology and taxonomy of *Spartina neyrautii *and *Spartina *alterniflora growing in France and Spain and comparison with Spartina townsendii and *Spartina anglica* Documents Phytosociologiques 1978 2 273 282

[B69] Ownbey M Natural hybridization and amphiploidy in the genus *Tragopogon* Am J Bot 1950 37 407 486 10.2307/2438023

[B70] Soltis DE Soltis PS Pires JC Kovarik A Tate JA Mavrodiev E Recent and recurrent polyploidy in *Tragopogon *(Asteraceae): cytogentic, genomic and genetic comparisons Biol J Linn Soc Lond 2004 8218079994

[B71] Kovarik A Pires JC Leitch AR Lim KY Sherwood AM Matyášek R Rocca J Soltis DE Soltis PS Rapid concerted evolution of nuclear ribosomal DNA in two *Tragopogon *allopolyploids of recent and recurrent origin Genetics 2005 169 2 931 944 10.1534/genetics.104.032839 15654116 PMC1449095

[B72] Matyášek R Tate JA Lim YK Srubarova H Koh J Leitch AR Soltis DE Soltis PS Kovarik A Concerted evolution of rDNA in recently formed *Tragopogon *allotetraploids is typically associated with an inverse correlation between gene copy number and expression Genetics 2007 176 4 2509 2519 10.1534/genetics.107.072751 17603114 PMC1950650

[B73] Hegarty MJ Jones JM Wilson ID Barker GL Coghill JA Sanchez-Baracaldo P Liu G Buggs RJ Abbott RJ Edwards KJ Development of anonymous cDNA microarrays to study changes to the *Senecio *floral transcriptome during hybrid speciation Mol Ecol 2005 14 8 2493 2510 10.1111/j.1365-294x.2005.02608.x 15969730

[B74] Mavrodiev E Soltis PS Baldini RM Gitzendanner MA Soltis DE Phylogeny of *Tragopogon porrifolius *L. (Asteraceae), a European native with intercontinental disjuncts Int J Plant Sci 2007 168 889 904 10.1086/518258

[B75] Bremer K Asteraceae: Cladistics and classification 1994 Portland: Timber Press

[B76] Borisova AG Shishkin BK Tragopogon Flora of USSR 29 1964 Moscow and Leningrad: Academy of the USSR 115 196

[B77] Ownbey M McCollum GD The chromosomes of *Tragopogon* Rodora 1954 56 7 21

[B78] Ownbey M McCollum GD Cytoplasmic inheritance and reciprocal amphiploidy in *Tragopogon* Am J Bot 1953 40 788 796 10.2307/2438276

[B79] Brehm BG Ownbey M Variation in Chromatographic patterns in the *Tragopogon dubius*-*pratensis*-*porrifolius *complex (Compositae) Am J Bot 1965 52 8 811 818 10.2307/2439762

[B80] Roose ML Gottlieb LD Genetic and biochemical consequences of polyploiy in *Tragopogon* Evolution 1976 30 818 830 10.2307/2407821 28563335

[B81] Soltis DE Soltis PS The dynamic nature of polyploid genomes Proc Natl Acad Sci USA 1995 92 18 8089 8091 10.1073/pnas.92.18.8089 7667249 PMC41101

[B82] Soltis DE Soltis PS Allopolyploid speciation in *Tragopogon*: Insights from chloroplast DNA Am J Bot 1989 76 1119 1124 10.2307/2444824

[B83] Cook LM Soltis PS Brunsfeld SJ Soltis DE Multiple independent formations of *Tragopogon *tetraploids (Asteraceae): evidence from RAPD markers Mol Ecol 1998 7 10 10.1046/j.1365-294x.1998.00453.x

[B84] Soltis PS Plunkett GM Novak SJ Soltis DE Genetic variation in *Tragopogon *species additional origin of the allotetraploids *T. mirus *and *T. miscellus* Am J Bot 1995 82 1329 1341 10.2307/2446255

[B85] Soltis PS Soltis DE The role of genetic and genomic attributes in the success of polyploids Proc Natl Acad Sci USA 2000 97 13 7051 7057 10.1073/pnas.97.13.7051 10860970 PMC34383

[B86] Tate JA Joshi P Soltis KA Soltis PS Soltis DE On the road to diploidization? Homoeolog loss in independently formed populations of the allopolyploid *Tragopogon miscellus *(Asteraceae) BMC Plant Biol 2009 9 80 10.1186/1471-2229-9-80 19558696 PMC2708164

[B87] Buggs RJA Doust AN Tate JA Koh J Soltis K Feltus FA Paterson AH Soltis PS Soltis DE Gene loss and silencing in *Tragopogon miscellus *(Asteraceae): comparison of natural and synthetic allotetraploids Heredity 2009 19277058 10.1038/hdy.2009.24

[B88] Tate JA Symonds VV Doust AN Buggs RJA Mavrodiev E Majure LC Soltis PS Soltis DE Synthetic polyploids of *Tragopogon miscellus *and *T. mirus *(Asteraceae): 60 years after Ownbey's discovery Am J Bot 2009 96 979 988 10.3732/ajb.0800299 21628250

[B89] Reineke A Lobmann S Gene expression changes in *Ephestia kuehniella *caterpillars after parasitization by the endoparasitic wasp *Venturia canescens *analyzed through cDNA-AFLPs J Insect Physiol 2005 51 8 923 932 10.1016/j.jinsphys.2005.04.007 15949813

[B90] Pathan AA Uma Devi K Vogel H Reineke A Analysis of differential gene expression in the generalist entomopathogenic fungus *Beauveria bassiana *(Bals.) Vuillemin grown on different insect cuticular extracts and synthetic medium through cDNA-AFLPs Fungal Genet Biol 2007 44 12 1231 1241 10.1016/j.fgb.2007.07.002 17723310

[B91] Wang J Lee JJ Tian L Lee HS Chen M Rao S Wei EN Doerge RW Comai L Chen ZJ Methods for genome-wide analysis of gene expression changes in polyploids Methods Enzymol 2005 395 570 596 full_text 15865985 10.1016/S0076-6879(05)95030-1 PMC1986650

[B92] Bennett RI Smith AG Use of a genomic clone for ribosomal RNA from *Brassica oleracea *in RFLP analysis of *Brassica *species Plant Mol Biol 1991 16 685 688 10.1007/BF00023432 1678285

[B93] O'Kane SL Schaal BA Al-Shehbaz IA The origins of *Arabidopsis suecica *as indicated by nuclear rDNA sequences Syst Bot 1996 21 559 566 10.2307/2419615

[B94] Skalicka K Lim KY Matyášek R Koukalova B Leitch A Kovaík A Rapid evolution of parental rDNA in a synthetic tobacco allotetraploid line Am J Bot 2003 90 988 996 10.3732/ajb.90.7.988 21659197

[B95] Skalicka K Lim KY Matyášek R Matzke M Leitch AR Kovarik A Preferential elimination of repeated DNA sequences from the paternal, Nicotiana tomentosiformis genome donor of a synthetic, allotetraploid tobacco New Phytol 2005 166 1 291 303 10.1111/j.1469-8137.2004.01297.x 15760371

[B96] Lin L-S Ho TD Harlan JR Rapid amplification and fixation of new restriction sites in the ribosomal DNA repeats in the derivatives of a cross between maize and *Tripsacum dactyloides* Devel Genetics 1985 6 101 112 10.1002/dvg.1020060204

[B97] Cluster PD Calderini O Pupilli F Crea F Fea Damiani The fate of ribosomal genes in three interspecific somatic hybrids of *Medicago sativa*: three different outcomes including the rapid amplification of new spacer-length variants Theor Appl Genet 1996 93 801 808 10.1007/BF00224079 24162411

[B98] Lim KY Souckova-Skalicka K Sarasan V Clarkson JJ CMWe A genetic appraisal of a new synthetic *Nicotiana tabacum *(Solanaceae) and the Kostoff synthetic tobacco Am J Bot 2006 93 875 883 10.3732/ajb.93.6.875 21642150

[B99] Pontes O Neves N Silva M Lewis MS Madlung A Comai L Viegas W Pikaard CS Chromosomal locus rearrangements are a rapid response to formation of the allotetraploid *Arabidopsis suecica *genome Proc Natl Acad Sci USA 2004 101 52 18240 18245 10.1073/pnas.0407258102 15604143 PMC539792

[B100] Kovarýk A Matyášek R Lim KY Skalicka K Koukalova B Knapp S Chase M Leitch AR Concerted evolution of 18-5.8-26S rDNA repeats in Nicotiana allotetraploids Biol J Linn Soc Lond 2004 82 615 625 10.1111/j.1095-8312.2004.00345.x

[B101] Guggisberg A Baroux C Grossniklaus U Conti E Genomic origin and organization of the allopolyploid *Primula egaliksensis *investigated by in situ hybridization Ann Bot (Lond) 2008 101 7 919 927 10.1093/aob/mcn026 PMC2710232 18308718

[B102] Lim KY Soltis DE Soltis PS Tate J Matyasek R Srubarova H Kovarik A Pires JC Xiong Z Leitch AR Rapid chromosome evolution in recently formed polyploids in *Tragopogon *(Asteraceae) PLoS ONE 2008 3 e3353 10.1371/journal.pone.0003353 18843372 PMC2556386

[B103] Liu B Segal G Vega JM Feldman M S A Isolation and characterization of chromosome-specific DNA sequences from a chromosome arm genomic library of common wheat Plant J 1997 11 959 965 10.1046/j.1365-313X.1997.11050959.x

[B104] Liu B Vega JM Segal G Abbo S Rodova H Feldman M Rapid genomic changes in newly synthesized amphiploids of *Triticum *and *Aegilops*. I. Changes in low-copy noncoding DNA sequences Genome 1998 41 272 277 10.1139/gen-41-2-272 9796102

[B105] Levy A Feldman M Genetic and epigenetic reprogramming of the wheat genome upon allopolyploidization Biol J Linn Soc Lond 2004 82 607 613 10.1111/j.1095-8312.2004.00346.x

[B106] Lim KY Soltis DE Soltis PS Tate J Matyášek R Srubarova H Kovarik A Pires JC Xiong Z Leitch AR Rapid chromosome evolution in recently formed polyploids in *Tragopogon *(Asteraceae) PLoS ONE 2008 3 e3353 10.1371/journal.pone.0003353 18843372 PMC2556386

[B107] Tarze A Deniaud A Le Bras M Maillier E Molle D Larochette N Zamzami N Jan G Kroemer G Brenner C GAPDH, a novel regulator of the pro-apoptotic mitochondrial membrane permeabilization Oncogene 2007 26 18 2606 2620 10.1038/sj.onc.1210074 17072346

[B108] Madlung A Masuelli RW Watson B Reynolds SH Davison J Comai L Remodeling of DNA methylation and phenotypic and transcriptional changes in synthetic *Arabidopsis *allotetraploids Plant Physiol 2002 129 2 733 746 10.1104/pp.003095 12068115 PMC161697

[B109] Preuss S Pikaard CS rRNA gene silencing and nucleolar dominance: insights into a chromosome-scale epigenetic on/off switch Biochim Biophys Acta 2007 1769 5-6 383 392 17439825 10.1016/j.bbaexp.2007.02.005 PMC2000449

[B110] Chen ZJ Genetic and epigenetic mechanisms for gene expression and phenotypic variation in plant polyploids Annu Rev Plant Biol 2007 58 377 406 10.1146/annurev.arplant.58.032806.103835 17280525 PMC1949485

[B111] Bachem CW Hoeven RS van der de Bruijn SM Vreugdenhil D Zabeau M Visser RG Visualization of differential gene expression using a novel method of RNA fingerprinting based on AFLP: analysis of gene expression during potato tuber development Plant J 1996 9 5 745 753 10.1046/j.1365-313X.1996.9050745.x 8653120

[B112] Adams KL Percifield R Wendel JF Organ-specific silencing of duplicated genes in a newly synthesized cotton allotetraploid Genetics 2004 168 4 2217 2226 10.1534/genetics.104.033522 15371349 PMC1448729

[B113] Comai L Genetic and epigenetic interactions in allopolyploid plants Plant Mol Biol 2000 43 2-3 387 399 10.1023/A:1006480722854 10999418

[B114] He P Friebe BR Gill BS Zhou J-M Allopolyploidy alters gene expression in the highly stable hexaploid wheat Plant Mol Biol 2003 52 401 414 10.1023/A:1023965400532 12856945

[B115] Konieczny A Ausubel FM A procedure for mapping *Arabidopsis *mutations using co-dominant ecotype-specific PCR-based markers Plant J 1993 4 2 403 410 10.1046/j.1365-313X.1993.04020403.x 8106085

[B116] Kim S Yoo M Albert VA Farris JS Soltis PS Soltis DE Phylogeny and diversification of B-function MADS-box genes in angiosperms: evolutionary and functional implications of a 260-million-year-old duplication Am J Bot 2004 91 2102 2118 10.3732/ajb.91.12.2102 21652358

[B117] Doyle JJ Doyle JL A rapid DNA isolation procedure for small quantities of fresh leaf tissue Phytochem Bull 1987 19 11 15

[B118] Sambrook J Russell DW Molecular cloning: a laboratory manual 2001 Cold Spring Harbor: Cold Spring Harbor Laboratory Press

