# Supplementary material for: Homeolog loss and expression changes in natural populations of the recently and repeatedly formed allotetraploid Tragopogon mirus (Asteraceae)
Source: BMC Genomics. 2010 Feb 8;11:97. doi: 10.1186/1471-2164-11-97 (PMC2829515; doi:10.1186/1471-2164-11-97)
Supplement: Additional file 1 — Supplementary Data. Homeologous loci and restriction enzymes examined in T. mirus with genomic and cDNA CAPS analyses. [file 1471-2164-11-97-S1.DOC]

**Additional file 1. Homeologous loci and restriction enzymes examined in *T. mirus* with genomic and cDNA anaylses**

| Putative Gene | Diagnostic restriction enzyme | Genomic fragment sizes (bp) | | cDNA fragment sizes (bp) | |
| --- | --- | --- | --- | --- | --- |
|  |  | *T. dubius* | *T. porrifolius* | *T. dubius* | *T. porrifolius* |
| *Fructose-biphosphate aldolase* |  | 397 | 454 | 303 | 360 |
| *Small GTP-binding protein* | BsmAI | 200 | 318 | 200 | 318 |
|  |  | 115 |  | 115 |  |
| *Thioredoxin M-type 1* | DdeI | 88 | 164 | 88 | 164 |
|  |  | 72 |  | 72 |  |
| *Transducin family protein* | - | 397 | 352 |  |  |
|  | NIaIII |  |  | 54 | 80 |
|  |  |  |  | 45 | 45 |
|  |  |  |  | 26 |  |
| *Gibberellin response modulator* | ApoI | 383 | 225 | 383 | 225 |
|  |  |  | 155 |  | 155 |
| *nuclear ribosomal DNA* | BstNI | 542 | 714 | 542 | 714 |
|  |  | 172 |  | 172 |  |
| *Expression protein 1* | PleI | 412 | 299 | 285 | 299 |
|  |  | 216 |  | 254 |  |
|  |  | 83 |  | 83 |  |
| *Conserved hypothetical protein* | HindII | 583 | 302 | 395 | 232 |
|  |  | 142 | 281 |  | 142 |
|  |  | 28 | 142 |  |  |
|  |  |  | 28 |  |  |
| *Cryptochrome* | AciI | 385 | 385 | 385 | 385 |
|  |  |  | 235 |  | 235 |
|  |  |  | 150 |  | 150 |
| *Glyceraldehyde-3-phosphate* | DelI | 378 | 606 | 278 | 504 |
| *dehydrogenase* |  | 220 |  | 220 |  |
| *Myosin heavy chain* |  | 520 | 430 | 520 | 430 |
| *Biotin synthase* | DdeI | 334 | 336 | 334 | 336 |
|  |  | 240 | 282 | 240 | 282 |
| *Peroxidase* | BstNI | 509 | 270 | 509 | 270 |
|  |  |  | 213 |  | 213 |
| *far-red impaired response* | NdeI | 295 | 360 | 365 | 326 |
| *protein* |  | 240 |  | 220 |  |
|  |  | 185 |  |  |  |
|  |  | 97 |  |  |  |
| *Glycosyl-transferase family 4* | Sau3AI | 430 | 380 | 345 | 300 |
|  |  |  |  |  | 82 |
| *Nucleic acid binding* | MboII | 720 | 500 |  |  |
|  |  |  | 284 |  |  |

**Additional file 1 (Continued)**

| Putative Gene | Diagnostic restriction enzyme | Genomic fragment sizes (bp) | | cDNA fragment sizes (bp) | |
| --- | --- | --- | --- | --- | --- |
|  |  | *T. dubius* | *T. porrifolius* | *T. dubius* | *T. porrifolius* |
| *RNA binding* | DraI | 380 | 520 |  |  |
|  |  | 180 |  |  |  |
| *porphyrin-oxidoreductase* | PleI | 400 | 396 |  |  |
|  |  | 112 |  |  |  |
|  |  | 86 |  |  |  |
| *LRR protein* | AluI | 296 | 136 |  |  |
|  |  | 224 | 108 |  |  |
|  |  | 124 |  |  |  |
| *Prenyltransferase* | NdeI | 296 | 398 |  |  |
|  |  | 99 |  |  |  |
| *Tetratricopeptide repeat protein* | MboII | 502 | 263 |  |  |
|  |  |  | 42 |  |  |
| *Adenine DNA glycosylase* | Aci I | 289 | 220 |  |  |
|  |  |  | 69 |  |  |
| *Poly-ubiquitin* | Sau3AI | 624 | 621 |  |  |
|  |  |  | 312 |  |  |
| *Leucine-rich repeat transmem-* | MaeII | 393 | 302 |  |  |
| *brane protein kinase* |  | 91 |  |  |  |
| *UDP-D-apiose-UDP-D-xylose* | PIeI | 415 | 512 |  |  |
| *synthetase* |  |  | 250 |  |  |
| *Protein phosphatase 2C family* | Nla III | 186 | 277 |  |  |
| *protein* |  | 94 |  |  |  |
| *Heat shock protein 70* | Sau3AI | 560 |  |  |  |
|  |  | 280 |  |  |  |
| *Glucosyl-transferase* | Sau3AI | 480 | 420 |  |  |
|  |  | 34 |  |  |  |
| *NADP/FAD oxidoreductase* | DdeI | 164 | 350 |  |  |
|  |  | 48 |  |  |  |
| *Hypothetical protein* | AciI | 365 | 581 |  |  |
|  |  | 215 |  |  |  |
