# Supplementary material for: Homeolog loss and expression changes in natural populations of the recently and repeatedly formed allotetraploid Tragopogon mirus (Asteraceae)
Source: BMC Genomics. 2010 Feb 8;11:97. doi: 10.1186/1471-2164-11-97 (PMC2829515; doi:10.1186/1471-2164-11-97)
Supplement: Additional file 2 — Supplementary Data. Genomic and cDNA CAPS analyses illustrating homeolog loss in a putative homolog of MYOSIN HEAVY CHAIN CLASS XI from multiple individuals from several populations of independent origin of T. mirus; also shown are the parental diploids, T. dubius and T. porrifolius. Tdu = T. dubius, Tm = T. mirus, Tpo = T. porrifolius. Arrows indicate missing homeologs. [file 1471-2164-11-97-S2.PPT]

## Slide 1
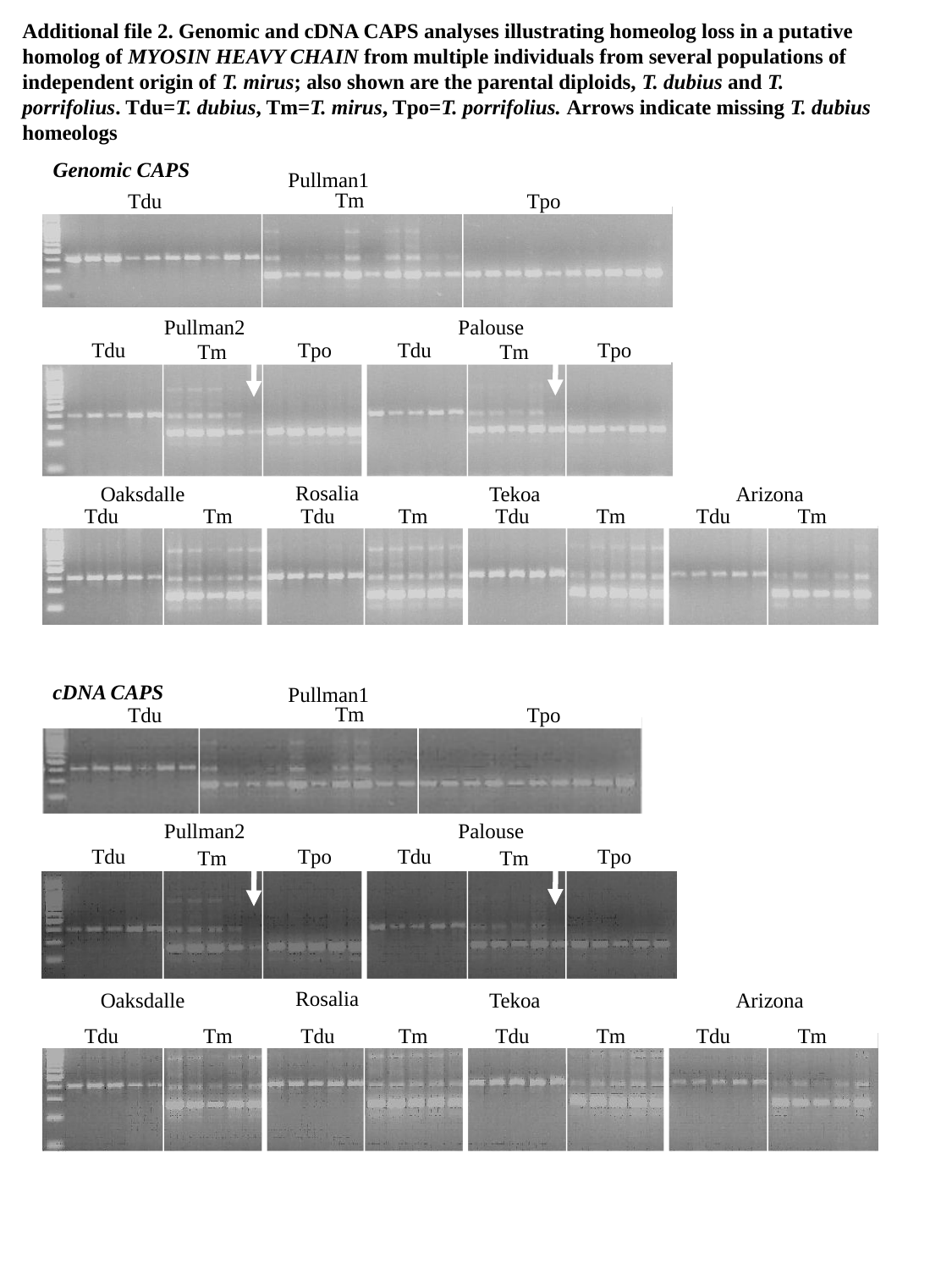

Additional file 2. Genomic and cDNA CAPS analyses illustrating homeolog loss in a putative homolog of MYOSIN HEAVY CHAIN from multiple individuals from several populations of independent origin of T. mirus; also shown are the parental diploids, T. dubius and T. porrifolius. Tdu=T. dubius, Tm=T. mirus, Tpo=T. porrifolius. Arrows indicate missing T. dubius homeologs
Genomic CAPS
Pullman1
Tm
Tdu
Tpo
Pullman2
Palouse
Tdu
Tpo
Tdu
Tpo
Tm
Tm
Rosalia
Oaksdalle
Tekoa
Arizona
Tdu
Tm
Tdu
Tm
Tdu
Tm
Tdu
Tm
cDNA CAPS
Pullman1
Tm
Tdu
Tpo
Pullman2
Palouse
Tdu
Tpo
Tdu
Tpo
Tm
Tm
Rosalia
Oaksdalle
Tekoa
Arizona
Tdu
Tm
Tdu
Tm
Tdu
Tm
Tdu
Tm
