# Supplementary material for: Homeolog loss and expression changes in natural populations of the recently and repeatedly formed allotetraploid Tragopogon mirus (Asteraceae)
Source: BMC Genomics. 2010 Feb 8;11:97. doi: 10.1186/1471-2164-11-97 (PMC2829515; doi:10.1186/1471-2164-11-97)
Supplement: Additional file 3 — Supplementary Data. Genomic and cDNA CAPS analyses of NUCLEAR RIBOSOMAL DNA, which exhibits silencing pattern in one plant of T. mirus from Tekoa (see red arrow). This plant is not expressing the homeolog of T. dubius. Tdu = T. dubius, Tm = T. mirus, Tpo = T. porrifolius. [file 1471-2164-11-97-S3.PPT]

## Slide 1
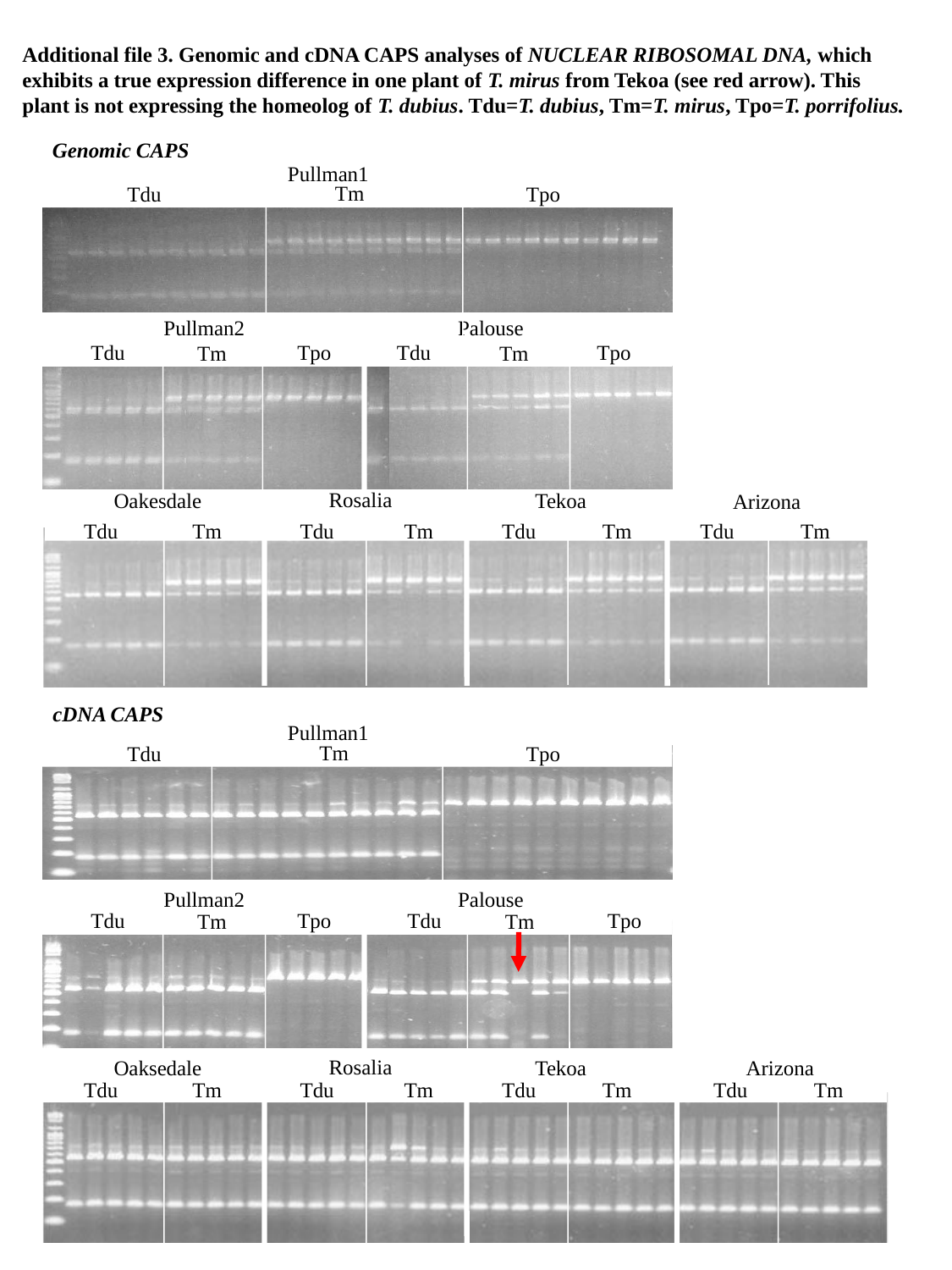

Additional file 3. Genomic and cDNA CAPS analyses of NUCLEAR RIBOSOMAL DNA, which
exhibits a true expression difference in one plant of T. mirus from Tekoa (see red arrow). This
plant is not expressing the homeolog of T. dubius. Tdu=T. dubius, Tm=T. mirus, Tpo=T. porrifolius.
Genomic CAPS
Pullman1
Tm
Tdu
Tpo
Pullman2
Palouse
Tdu
Tpo
Tdu
Tpo
Tm
Tm
Rosalia
Oakesdale
Tekoa
Arizona
Tdu
Tm
Tdu
Tm
Tdu
Tm
Tdu
Tm
cDNA CAPS
Pullman1
Tm
Tdu
Tpo
Pullman2
Palouse
Tdu
Tpo
Tdu
Tpo
Tm
Tm
Rosalia
Oaksedale
Tekoa
Arizona
Tdu
Tm
Tdu
Tm
Tdu
Tm
Tdu
Tm
