# Supplementary material for: Homeolog loss and expression changes in natural populations of the recently and repeatedly formed allotetraploid Tragopogon mirus (Asteraceae)
Source: BMC Genomics. 2010 Feb 8;11:97. doi: 10.1186/1471-2164-11-97 (PMC2829515; doi:10.1186/1471-2164-11-97)
Supplement: Additional file 4 — Supplementary Data. Genomic and cDNA CAPS analyses for 15 candidate genes from Tragopogon F1 hybrids and their porgenitors. Tdu = T. dubius(2611-11, Pullman, WA), Tpo = T. porrifolius (2613-24, Pullman, WA). [file 1471-2164-11-97-S4.PPT]

## Slide 1
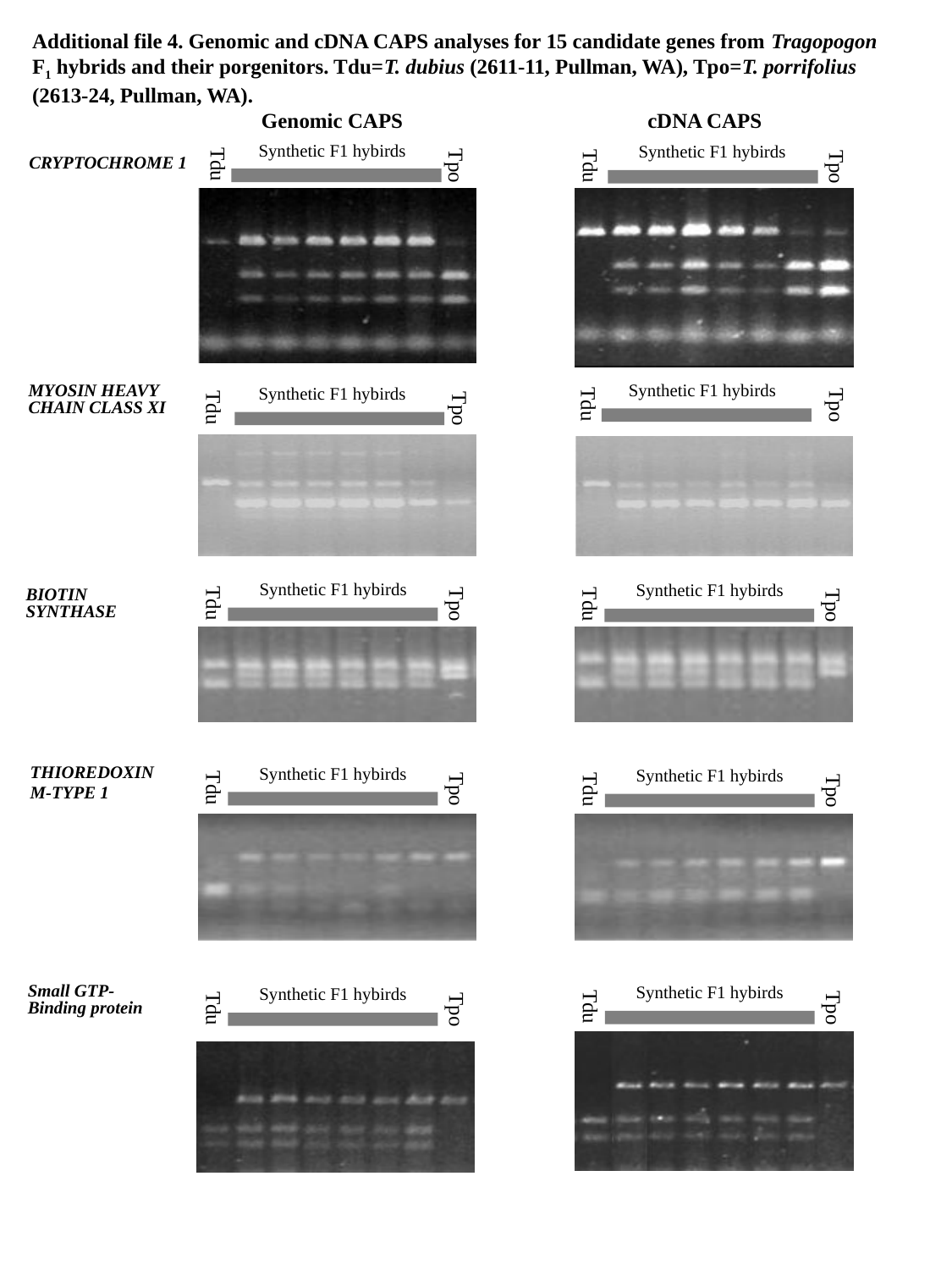

Additional file 4. Genomic and cDNA CAPS analyses for 15 candidate genes from Tragopogon F1 hybrids and their porgenitors. Tdu=T. dubius (2611-11, Pullman, WA), Tpo=T. porrifolius (2613-24, Pullman, WA).
Genomic CAPS
cDNA CAPS
Synthetic F1 hybirds
Synthetic F1 hybirds
Tdu
Tpo
Tdu
Tpo
CRYPTOCHROME 1
Synthetic F1 hybirds
Synthetic F1 hybirds
MYOSIN HEAVY
CHAIN CLASS XI
Tdu
Tpo
Tdu
Tpo
Synthetic F1 hybirds
Synthetic F1 hybirds
Tdu
BIOTIN SYNTHASE
Tpo
Tdu
Tpo
THIOREDOXIN
M-TYPE 1
Synthetic F1 hybirds
Synthetic F1 hybirds
Tdu
Tpo
Tdu
Tpo
Synthetic F1 hybirds
Synthetic F1 hybirds
Small GTP-
Binding protein
Tdu
Tpo
Tdu
Tpo

## Slide 2
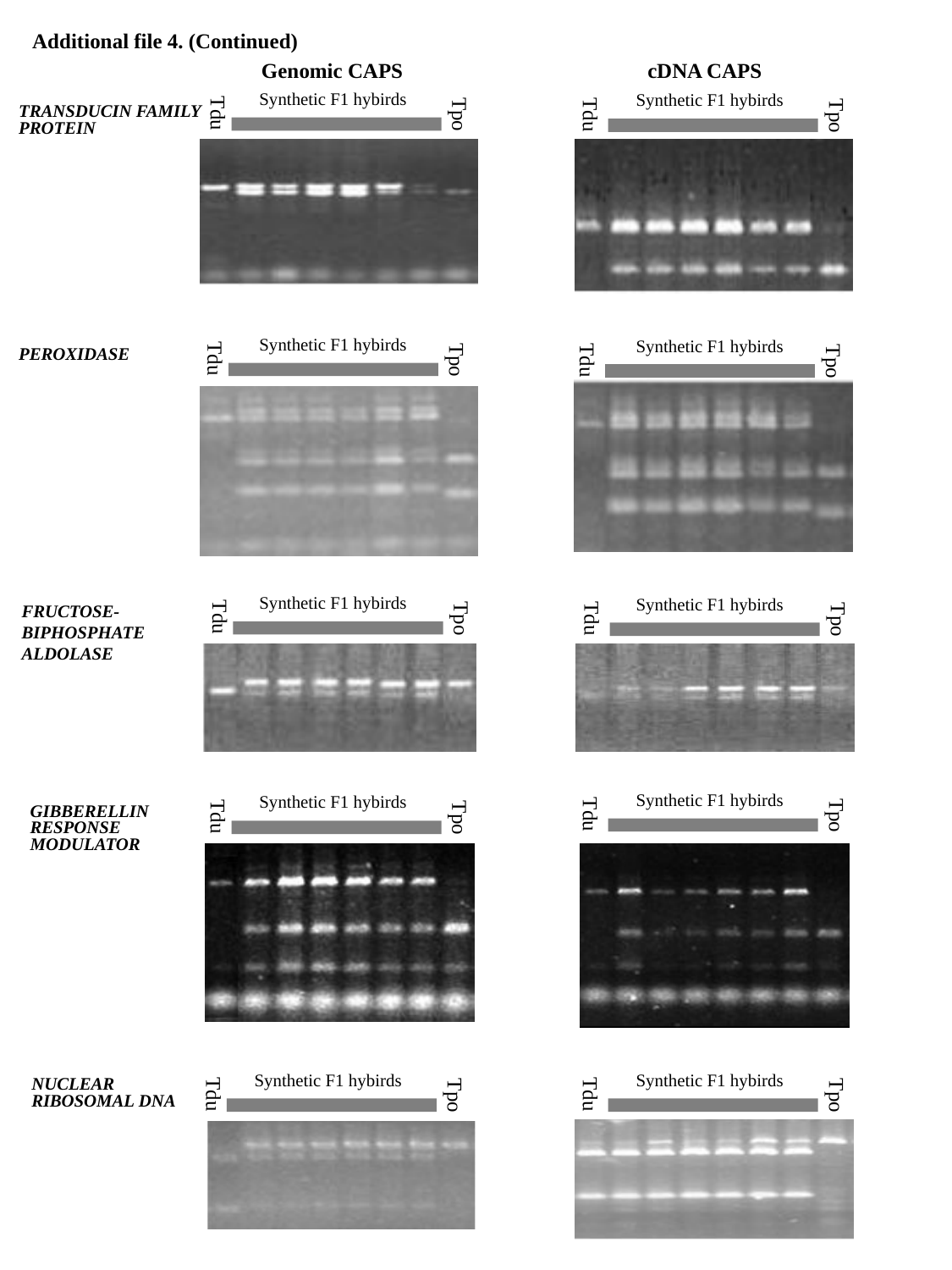

Additional file 4. (Continued)
Genomic CAPS
cDNA CAPS
Synthetic F1 hybirds
Synthetic F1 hybirds
Tdu
Tpo
Tdu
Tpo
TRANSDUCIN FAMILY PROTEIN
Synthetic F1 hybirds
Synthetic F1 hybirds
Tdu
Tpo
Tdu
Tpo
PEROXIDASE
Synthetic F1 hybirds
Synthetic F1 hybirds
FRUCTOSE-BIPHOSPHATE ALDOLASE
Tdu
Tpo
Tdu
Tpo
Synthetic F1 hybirds
Synthetic F1 hybirds
Tdu
Tpo
Tdu
Tpo
GIBBERELLIN
RESPONSE
MODULATOR
Synthetic F1 hybirds
Synthetic F1 hybirds
NUCLEAR
RIBOSOMAL DNA
Tdu
Tdu
Tpo
Tpo

## Slide 3
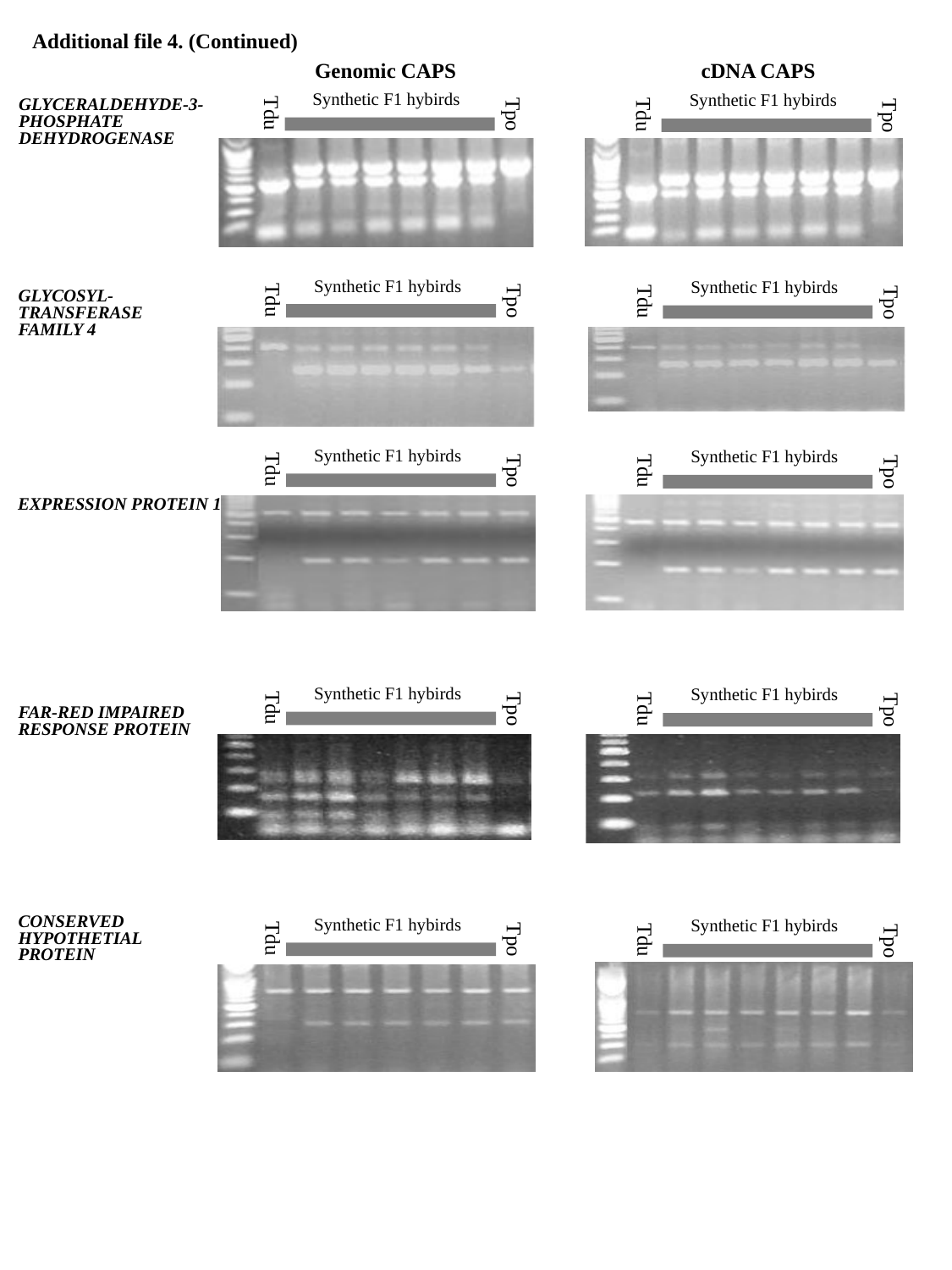

Additional file 4. (Continued)
Genomic CAPS
cDNA CAPS
Synthetic F1 hybirds
Synthetic F1 hybirds
Tdu
GLYCERALDEHYDE-3-
PHOSPHATE
DEHYDROGENASE
Tpo
Tdu
Tpo
Synthetic F1 hybirds
Synthetic F1 hybirds
Tdu
Tpo
Tdu
Tpo
GLYCOSYL-
TRANSFERASE
FAMILY 4
Synthetic F1 hybirds
Synthetic F1 hybirds
Tdu
Tpo
Tdu
Tpo
EXPRESSION PROTEIN 1
Synthetic F1 hybirds
Synthetic F1 hybirds
Tdu
Tpo
Tdu
Tpo
FAR-RED IMPAIRED
RESPONSE PROTEIN
Synthetic F1 hybirds
CONSERVED HYPOTHETIAL PROTEIN
Synthetic F1 hybirds
Tdu
Tpo
Tdu
Tpo
