# Supplementary material for: Homeolog loss and expression changes in natural populations of the recently and repeatedly formed allotetraploid Tragopogon mirus (Asteraceae)
Source: BMC Genomics. 2010 Feb 8;11:97. doi: 10.1186/1471-2164-11-97 (PMC2829515; doi:10.1186/1471-2164-11-97)
Supplement: Additional file 5 — Supplementary Data. Primer combination for selective amplification and used in cDNA-AFLP analyses. Asterisk indicates primers used in expanded study. [file 1471-2164-11-97-S5.DOC]

**Additional file 5. Primer combination for selective amplification and used in cDNA-AFLP analyses. Asterisk indicates primers used in expanded study.**

|  | ***EcoRI* primer** | | | | | | |
| --- | --- | --- | --- | --- | --- | --- | --- |
|  | **E-AA** | **E-AC** | **E-AG** | **E-AT** | **E-TA** | **E-TC** | **E-TG** |
| ***MseI***  **primer** |  | M-CAA* | M-CAA* | M-CAA |  |  | M-CAA* |
|  | M-CAC* | M-CAC* | M-CAC* | M-CAC | M-CAC | M-CAC | M-CAC |
|  | M-CAG |  | M-CAG | M-CAC* | M-CAG* | M-CAG | M-CAG |
|  | M-CAT* |  | M-CAT* | M-CAG | M-CAT* | M-CAT* |  |
|  |  | M-CTA | M-CTA* | M-CTA* |  |  |  |
|  | M-CTC |  | M-CTC | M-CTC |  |  |  |
|  |  |  | M-CTG | M-CTG |  |  |  |
|  | M-CTT* | M-CTT* | M-CTT* | M-CTT* | M-CTT* | M-CTT* | M-CTT* |
