# Supplementary material for: Homeolog loss and expression changes in natural populations of the recently and repeatedly formed allotetraploid Tragopogon mirus (Asteraceae)
Source: BMC Genomics. 2010 Feb 8;11:97. doi: 10.1186/1471-2164-11-97 (PMC2829515; doi:10.1186/1471-2164-11-97)
Supplement: Additional file 6 — Supplementary Data. Primer. [file 1471-2164-11-97-S6.DOC]

**Additional file 6. Primer information used in genomic and cDNA CAPS analyses**

| Putative Gene | Genbank accession numbers | Primer name | Primer sequence (5’ to 3’) |
| --- | --- | --- | --- |
| *Adenine DNA glycosylase* | *T .dubius-* DQ267228 | ADGY-F1 | GGTGCATAAATCAGCACAACA |
|  | *T. porrifolius-*GU354166 | ADGY-R1 | ACAAACCTACCCTCCCCATC |
| *Fructose-biphosphate* | *T. dubius*-DQ267230 | BFRUCT4-F1 | GGAAGACCTTGATTGATCGG |
| *aldolase* | *T. porrifolius-*GU354167 | BFRUCT4-R1 | AAGGATGTTGTGGTGGAAGC |
| *Small GTP-binding protein* | *T. dubius-* DQ267232 | GTPB-F1 | CCTCTCTCTACAATTCCGGC |
|  | *T. porrifolius-*GU354168 | GTPB-R1 | TACGGCGACTGATGTCGTAA |
| *Leucine-rich repeat Transmem-* | *T. dubius-* DQ267234 | LTR2-F1 | GGGTTCTGTTACGAGCAAGG |
| *brane protein kinase* | *T. porrifolius-*GU354169 | LTR2-R1 | TGAAGTATTCGGGATCCATAT |
| *Protein phosphatase 2C family* | *T. dubius-*DQ267222 | PP2C-F1 | CTGGAAAACCAAATACCCGA |
| *protein* | *T. porrifolius-*GU354170 | PP2C-R1 | ATCTTCAGACCCCACCACAG |
| *Transducin family protein* | *T. dubius-* DQ267255 | TDRC-F2 | AAAGCCGAGGGTAAATCAGC |
|  | *T. porrifolius-*GU354171 | TDRC-R2 | TCGAGGCCTTGAATGTTTTT |
| *Thioredoxin M-type 1* | *T. dubius-* DQ267221 | THIOR-F1 | AATCAGAAGCATCCCGACTG |
|  | *T. porrifolius-*GU354172 | THIOR-R1 | CACAATCTTTTTGTGAAATGCAA |
| *Poly-ubiquitin* | *T. dubius-* DQ267236 | UBQ4-F1 | TCGACTCTCCACCTGGTTCT |
|  | *T. porrifolius-*GU354173 | UBQ4-R1 | CAGACATCACCACCACGAAG |
| *Glyceraldehyde-3-phosphate* | *T. dubius-*GU354174 | G3PDH-F1 | TTTGGAATTGTTGAGGGTCTC |
| *dehydrogenase* | *T. porrifolius-*GU354175 | G3PDH-R1 | TCACCCACAAAGTCAGTGGA |

**Additional file 6. (Continued)**

| Putative Gene | Genbank accession numbers | Primer name | Primer sequence (5’ to 3’) |
| --- | --- | --- | --- |
| *Myosin heavy chain* | *T. dubius-*GU354176 | MHC-F1 | CGACACGGAATATAGCATCC |
|  | *T. porrifolius-*GU354177 | MHC-R1 | GGATAAAGTGATGCTCATATGG |
| *Gibberellin reponse* | *T. dubius-*GU354178 | RGA-F1 | CGTCCACGTCGTTGATTTCA |
| *modulator* | *T. porrifolius-*GU354179 | RGA-R1 | GATTCGATTCCTGTTCCACG |
| *Nuclear ribosomal DNA* | *T. dubius-*AM493993 | nrDNA-F1 | GCGCTACACTGATGTATTCAACG |
|  | *T. porrifolius-*AM493994 | nrDNA-R1 | CGCAACTTGCGTTCAAAAACTCGA |
| *Heat shock protein 70* | *T. dubius-*GU354180 | HS-F1 | CTAACGACAAGGGTAGACTATC |
|  |  | HS-R1 | GGATCACATGCACATTAAGTGC |
| *Porphyrin oxidoreductase* | *T. dubius-*FJ708516 | AG_194F | TYTGYCGCAAACGCTGTC |
|  | *T. porrifolius-*GU354181 | AG_597R | TCWTCTTGAAAWGCTTGAACTCC |
| *NADP/FADoxidoreductase* | *T. dubius-*FJ708504 | LacSing3F | AGGCAAAGCACCTTCAAAGA |
|  | *T. porrifolius-*GU354182 | LacSing3R | CTTCCACTGCCAGCTTTTTC |
| *Biotin synthase* | *T. dubius-FJ708506* | LacSing4F | GGTCCGAGAACTGATTGGAA |
|  | *T. porrifolius-*GU354183 | LacSing4R | ATTGGGGGCAATATGAACAA |
| *Tetratricopeptide repeat* | *T. dubius*GU354184 | TPR-F1 | ATTCAGAGGAAGCCATCAAG |
| *protein* | *T. porrifolius-*GU354185 | TPR-R1 | GGCTTTTTCAATCCTGTTAATTC |
| *RNA binding* | *T. dubius-FJ708502* | AB3156F | TYACTCAYGTYTCAMGAGGATTTG |
|  | *T. porrifolius-*GU354186 | AB4248R | AGTAATAACCAGATGCTTCATCCC |

**Additional file 6. (Continued)**

| Putative Gene | Genbank accession numbers | Primer name | Primer sequence (5’ to 3’) |
| --- | --- | --- | --- |
| *LRR protein* | *T. dubius* -GU354187 | LRR-F1 | GCACGAGGCCAGGAAGTAG |
|  | *T. porrifolius-*GU354188 | LRR-R1 | ATCCACAATCGACTATGTTTG |
| *Prenyl transferase* | *T. dubius-*FJ708514 | AK_1074F | ARCCWGTMACTTGGCCTCC |
|  | *T. porrifolius-*GU354189 | AK_1461R | ATKGCTCCMGAWGGAATTGG |
| *Cryptochrome 1* | *T. dubius-*FJ770377 | Cry1-F1 | CTAAAACTCGTCCCACTAGAAG |
|  | *T. porrifolius-*GU354190 | Cry1-R1 | GGAATGGAAGAAGGACTCGG |
| *Glycosyltransferase family 4* | *T. dubius-*GU354191 | GlyTr-F1 | GTTTAAATATCCGAGCACCCC |
|  | *T. porrifolius-*GU354192 | GlyTr-R1 | ACGTGACTTGTTTGAGCTTC |
| *Glucosyl transferase* | *T. dubius-*GU354193 | Glu-F1 | AAAAGGAAGAGTTACTTCATGG |
|  | *T. porrifolius-*GU354194 | Glu-R1 | CCTCCAAACACCTCTTGATC |
| *Expression Protein1* | *T. dubius-*GU354195 | EP-F1 | AAAAGATTAGTTAGCTCTTGC |
|  | *T. porrifolius-*GU354196 | EP-R1 | GCTTCAAGGTAACGCCGTTT |
| *Hypothetical Protein* | *T. dubius-*GU354197 | AP-F1 | TTGCAATTACGATTTACAC |
|  | *T. porrifolius-*GU354198 | AP-R1 | GGCATACAGTGTCTGAAACG |
| *Conserved hypothetical* | *T. dubius-*GU354205 | PA-F1 | GGTAGTGCTGCATTTGCATG |
| *protein* | *T. porrifolius-*GU354206 | PA-R1 | TCTTGTTGCGAATGAGACCC |
| *UDP-D-apiose-UDP-D-xylose* | *T. dubius-*GU354199 | UDX-F | TCTGATCTTCCCAAGTCCAG |
| *synthetase* | *T. porrifolius-*GU354200 | UDX-R | AAAACAGCAGCACCTTACCC |

**Additional file 6. (Continued)**

| Putative Gene | Genbank accession numbers | Primer name | Primer sequence (5’ to 3’) |
| --- | --- | --- | --- |
| *Peroxidase* | *T. dubius-*GU354201 | Pero-F | TAATGTTGGCAGTGATGGTG |
|  | *T. porrifolius-*GU354202 | Pero-R | GGAACGTTATATTGTGGTCC |
| *far-red impaired response* | *T. dubius-*GU354203 | FrirLa-F | TGGGTCAAAAGTAATCTGGATTC |
| *Protein* | *T. porrifolius-*GU354204 | FRIR-R | AGCTTAGTCATGACGACATTG |
| *Nucleic acid binding* | *T. dubius-FJ708510* | LAC7FAD1_253F | WTGGGAAGAAGGMGAAGG |
|  | *T. porrifolius-*GU354207 | LAC7RAD_688R | TTATCRAACTGWGTWGTATCAAATGCC |
